# Stromal-based proteome data improve stratification of hormone receptor-positive breast cancer

**DOI:** 10.1038/s41523-026-00943-y

**Published:** 2026-04-09

**Authors:** Kenneth Finne, Silje Kjølle, Magdalena Ríos Romero, Even Birkeland, Heidrun Vethe, Sura Aziz, Gøril Knutsvik, Elisabeth Wik, Linda Sofie Lindström, Lars A. Akslen

**Affiliations:** 1 Centre for Cancer Biomarkers CCBIO, Department of Clinical Medicine, Section for Pathology, University of Bergen, Bergen, Norway; 2 Department of Oncology and Pathology, Karolinska Institutet, Stockholm, Sweden; 3 Breast Center, Karolinska Comprehensive Cancer Center, Karolinska University Hospital, Stockholm, Sweden; 4 Department of Pathology, Haukeland University Hospital, Bergen, Norway

**Keywords:** Biomarkers, Cancer, Computational biology and bioinformatics, Oncology

## Abstract

Breast cancers are biologically and clinically diverse. While large-scale gene expression analyses have enabled epithelial-centered molecular classifications, studies of the tumor microenvironment (TME) remain limited, especially at the proteome level using tissue-specific resolution. By laser capture microdissection and mass spectrometry-based proteomics followed by unsupervised clustering of the stromal proteome, we discovered three patient subgroups. The largest cluster revealed the most discrete representation of stromal proteins, including a 35-protein (35P) panel linked to extracellular matrix biology, tumor progression programs, and increased abundance of tumor-associated macrophages (TAM) by single-cell profiling. Clinical validation of 35P, using whole tissue protein and mRNA values from different cohorts of ER+/HER2− breast cancer, including a large randomized controlled trial (STO), identified that more aggressive (or ‘high-grade’) stromal features were independent of current molecular subtypes. The 35P stromal panel may reflect important clinical information by improving patient stratification beyond current epithelial-based classification of breast cancer.

## Introduction

Breast cancers are complex ecosystems of epithelial tumor cells and microenvironment (TME) components, and large-scale gene expression and genetic analyses have provided a basis for molecular classifications. The following main subtypes, reflecting tumor cell phenotypes, are now used in clinical practice: luminal-A, luminal-B, HER2-enriched, and triple-negative tumors^1^. The potential of the tumor microenvironment (TME) to add clinically useful information is not clear, although current knowledge supports a more active role of the TME in tumor progress and therapy response than previously thought.

A number of studies have shown that heterogeneity is present within the established tumor subtypes^2,3^, and some of this might be attributed to TME differences. We here examined the breast cancer proteome using separate samples of laser microdissected epithelial and stromal tumor tissues, in contrast to whole tissue ‘omics’ used in most other studies^1,2,4–9^. We aimed to perform a proteomics mapping with particular attention to variability in the stromal proteome and clinical correlates. Thus, we asked whether TME-based protein patterns might capture additional information independent of the current epithelial-based classification. Our findings suggest that stromal data can improve breast cancer stratification, in particular among assumed low-grade tumors, with potential impact on current management.

## Results

### The proteomes of breast cancer stroma and tumor cells show different patterns

Luminal A, luminal B, and basal-like subtypes were assigned by experienced pathologists using standard immunohistochemical (IHC) markers. Tumor epithelial cells and tumor stroma from 24 FFPE breast cancers (12 basal-like and 12 luminal-like; Supplementary Table 1; HER2+ cases were not included) were separated by laser capture microdissection, followed by analysis of the extracted proteomes using label-free MS-based shotgun proteomics. We quantified 4157 and 2150 proteins from microdissected tumor epithelium and tumor stroma, respectively.

**Fig. 1:**
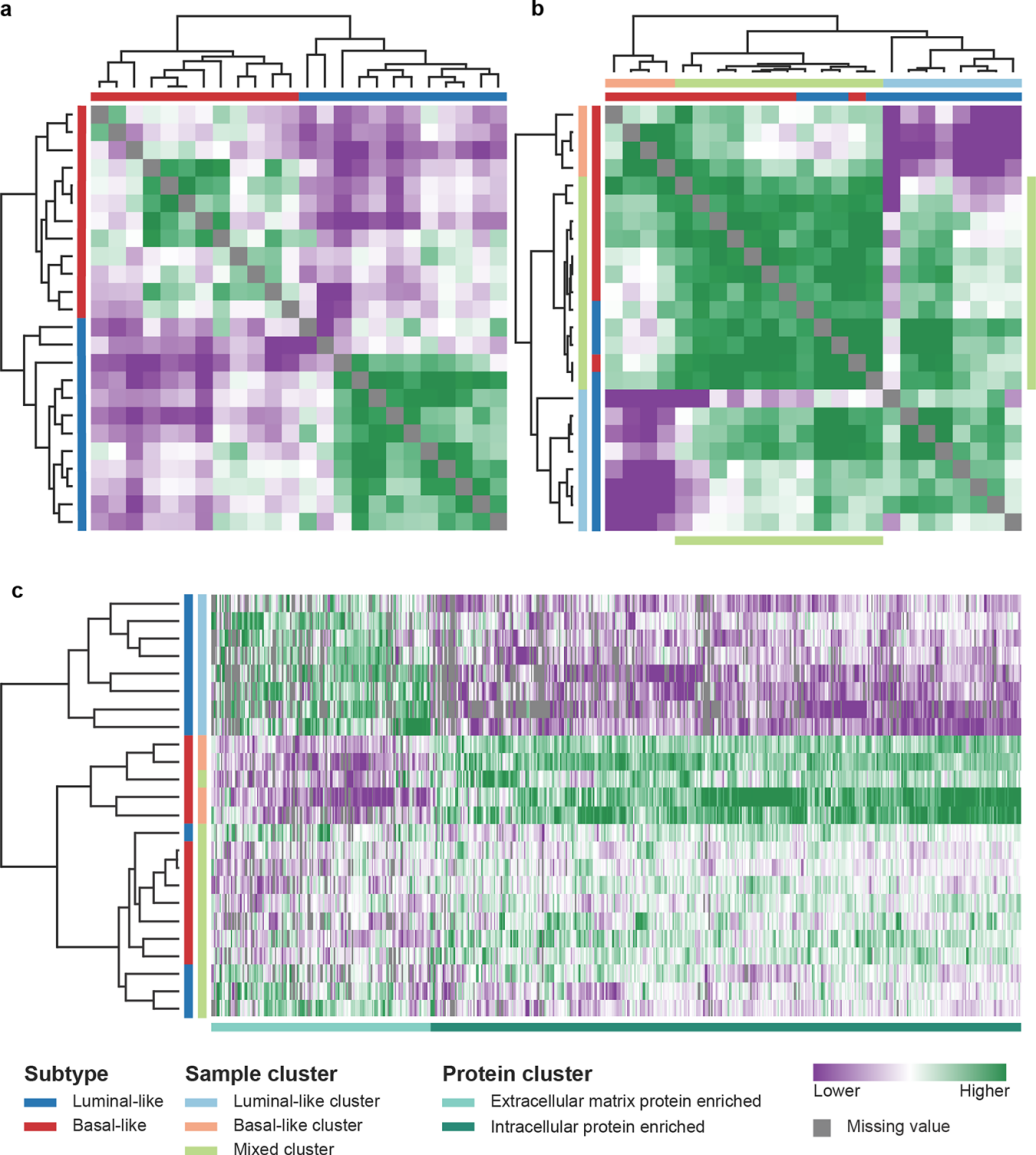
Proteomic correlation patterns in tumor epithelium and tumor stroma. **a** Unsupervised hierarchical clustering of tumor epithelial samples shows clear separation of basal‑like and luminal‑like tumors. **b** Tumor stroma samples cluster into three groups (basal‑like, luminal‑like, and mixed) independent of epithelial subtype. **c** Stromal proteins differing between the three stromal clusters (ANOVA) are shown by hierarchical clustering, highlighting ECM‑ and intracellular‑enriched groups.

Protein expression correlation analysis was performed to identify similarities between samples. By plotting the global correlations as a heatmap with unsupervised clustering, the tumor epithelium samples formed two distinct clusters that perfectly grouped the luminal-like and basal-like subtypes (Fig. 1a). The tumor stroma samples, however, generated three clusters: a “basal-like” cluster (*n* = 4), a “luminal-like” cluster (*n* = 8), and a mixed cluster (*n* = 12), the latter consisting of both luminal-like and basal-like samples (Fig. 1b). For comparison, whole tissue proteomics (*n* = 46) gave a mixed pattern with no distinct subgroups by unsupervised clustering (Supplementary Fig. 1). This pattern for the whole tissue proteome remained also after down-sampling the data to only include proteins found in the tumor stroma compartment (based on LCM data).

As the correlation analysis of microdissected tumor stroma samples did not simply reflect the epithelial-based subtypes, we further explored the clustering of stromal samples. The three stromal clusters (Fig. 1b) were compared (by ANOVA), and we found a significant difference in the ratio between extracellular matrix proteins and intracellular proteins between the basal-like and luminal-like clusters, with more intracellular proteins in the basal-like samples, suggesting a higher stromal cellularity (Fig. 1c and Supplementary Fig. 2). This difference appeared to contribute to the clustering of stromal tissues. The mixed cluster showed an intermediate stromal profile, with moderate levels of both intra- and extracellular proteins. Taken together, the observed patterns may reflect differences in cell-to-ECM ratios between the three stromal clusters.

To investigate whether the three clusters from Fig. 1c are associated with differences in immune cell content, we scored the presence of immune cell infiltration (TILs; score 1–4; increasing density of immune cells) using parallel HE-slides. The basal-like cluster had higher levels of immune cells (average score of 3.0), compared to the mixed cluster (average score of 1.8; Mann–Whitney *U*-test *p* = 0.049 versus basal-like cluster), and the luminal-like cluster (average score of 1.4, *p* = 0.008 versus basal-like cluster; *p* = 0.24 versus mixed cluster; Supplementary Fig. 3a). These differences were supported by imaging mass cytometry (IMC) single-cell quantification (using markers for T-cells, B-cells, and macrophages) on the same cases (Supplementary Fig. 3b).

The mixed stromal cluster consisted of both basal-like and luminal-like cases. The strong correlation between these samples (Fig. 1b) suggests that the stromal composition was relatively more similar between samples in this cluster. Thus, stromal tissues from the mixed cluster were selected for further analyses, assuming that this approach would be suitable for detecting novel stromal-based traits and not only reflect known variations in immune cell content between the three subgroups or between epithelial-based subtypes.

Since we focused on case differences based on the stromal compartment, we limited our investigation to proteins that were differentially abundant only in the tumor stroma, i.e., not also in the tumor epithelial compartment. Therefore, proteins that were differentially abundant between basal-like and luminal-like cases based on the tumor epithelial fraction (by two-sided Student’s *t* test, *p* < 0.05, no fold change cut-off) were subtracted from the list of differentially abundant proteins in stromal samples (two-sided Student’s *t* test, *p* < 0.05, fold change >1.5). Subsequently, we identified 35 proteins (35P) that remained significantly different between basal-like and luminal-like tumors in the stromal tissue fraction from the mixed patient cluster (Supplementary Table 2).

### The 35-protein stromal panel relates to extracellular components and single-cell composition of the tumor microenvironment

To investigate the 35P stromal protein signature in more detail, we performed a protein-protein interaction analysis (STRING) and found few intra-signature protein connections (Supplementary Fig. 4). Ten of the 35 proteins were components of the ECM, and the signature was overrepresented by proteins associated with “extracellular matrix” (such as CTSZ, MMP2 and PXDN; FDR < 0.001), “basement membrane” (such as AGRN, NID1 and VWA1; FDR = 0.008) and “extracellular exosomes” (such as IDH2, PIP and VAT1; FDR = 0.025; Supplementary Table 3).

Using an additional approach to bypass the proteome differences introduced by varying levels of immune cells, we re-analyzed all 24 microdissected stromal samples using an ECM database (Core Matrisome^10^). The differences in core ECM proteins between basal-like and luminal-like subtypes, within the mixed stromal cluster, were only moderate (8 significantly different proteins; higher abundance in basal-like samples: NID1, VWA1, COL12A1, AEBP1, and LAMB2; lower abundance in basal-like samples: COL1A1, VCAN, COL3A1; ranked by *p* value). Two proteins overlapped with the 35P signature (NID1 and VWA1). Of note, NID1 and VWA1 had the highest positive fold change when comparing basal-like and luminal-like cases in both the Core Matrisome analysis (4.4 and 3.7, respectively) and in the full proteome analysis (5.6 and 3.4, respectively).

**Fig. 2:**
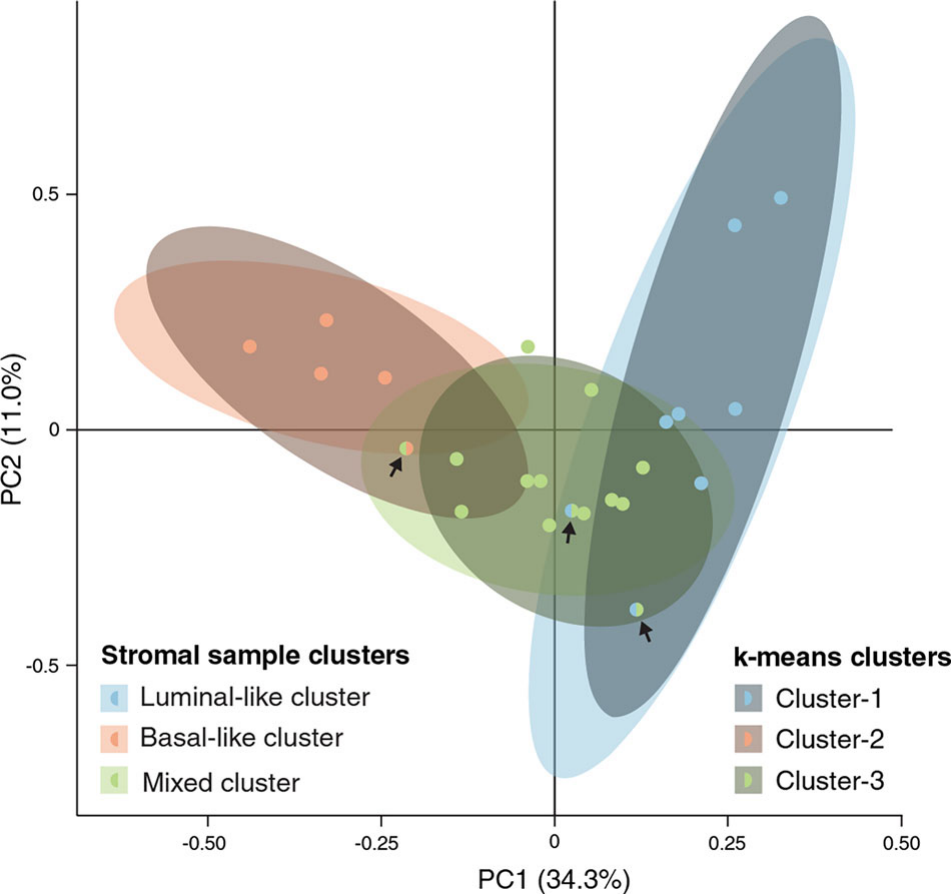
PCA of the core matrisome reflects stromal proteomic clusters. PCA of the core-matrisome separates stroma into three clusters that largely mirror the three stromal sample clusters identified by the full‑proteome analysis (arrows denote the three discordant samples).

We then used k-means clustering (*k* = 3) to group the stroma samples into three clusters to investigate how these aligned with our stromal sample clusters from Fig. 1b; clusters from these two analyses were highly similar (Fig. 2). This indicates that the matrisome proteins are relevant to the clustering from the full-proteome analysis.

Next, we asked whether 35P was also related to the single-cell composition in the stromal compartment. Data from Wu et al.^11^ (accessed through singlecell.broadinstitute.org) indicate that the 35P proteins associate with several stromal cell types, including cancer-associated fibroblasts, endothelial cells, perivascular-like cells, and macrophages (Supplementary Fig. 5).

**Fig. 3:**
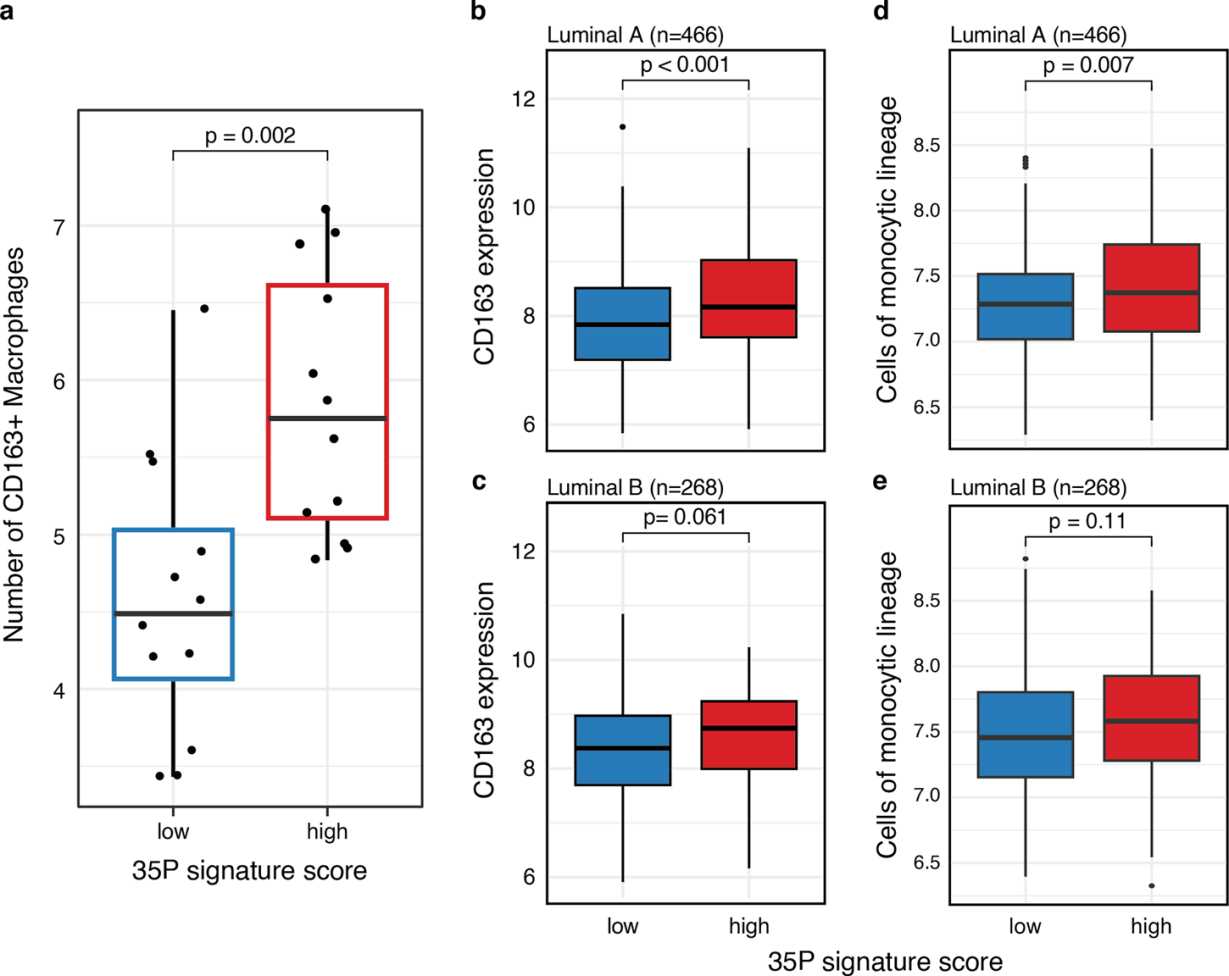
High 35P signature score is associated with increased levels of macrophages. **a** In-house imaging mass cytometry (IMC) data show that higher 35P score (proteomic data on the same cases; cutoff: median) is associated with higher number of CD163-positive macrophages (*p* = 0.002, *n* = 24). **b**, **c** Higher CD163-expression is associated with high 35P in patients with luminal A breast cancer (*p* < 0.001, *n* = 466), but not luminal B (*p* = 0.061, *n* = 268). **d**, **e** MCP counter analysis indicates higher counts of ‘cells of monocytic lineage’ in the 35P-high group in patients with luminal A breast cancer (*p* = 0.007, *n* = 466); no difference is observed in luminal B tumors (*p* = 0.11, *n* = 268). Cutoff for panels b-e was the upper quartile. *p* values were calculated using Mann–Whitney *U*-test. Box plots show the median (center line), interquartile range (box), and whiskers extending to 1.5 × the IQR, with points beyond the whiskers representing outliers. MCP cell deconvolution by Microenvironment Cell Populations.

Using in-house imaging mass cytometry (IMC)-based single-cell data from the 24 cases also applied for MS proteomics and 35P discovery, we found that high 35P score was associated with increased numbers of CD163+ macrophages (*p* = 0.002, Mann–Whitney *U*-test; Fig. 3a). Also, high 35P score was associated with increased CD163 gene expression levels (*p* < 0.001, Mann–Whitney *U*-test; Fig. 3b, c) and higher levels of macrophages by IMC (*p* = 0.037, GLM likelihood ratio test) in patients with luminal A breast cancer the METABRIC Discovery cohort^12,13^. Similarly, using cell deconvolution^14^, patients with luminal A breast cancer had higher levels of cells with monocytic lineage (*p* = 0.007; Mann–Whitney *U*-test^14^; Fig. 3d, e). In contrast, no significant differences were observed in luminal B breast cancer.

To investigate a link between macrophage count and patient outcome in luminal A breast cancer, we explored the IMC data established on the METABRIC cohort and reported by Danenberg et al.^13^, and we found that macrophage-high cases (by upper quartile) were associated with worse outcome (Log-rank *p* = 0.036; Supplementary Fig. 6).

Taken together, our findings indicate that the 35P signature relates to both extracellular matrix proteins and in particular macrophage content in the tumor microenvironment. Furthermore, these data suggest an association between high stromal 35P status and macrophage enrichment, and a link between high macrophage count and reduced patient survival in luminal A breast cancer.

### The stromal proteome shows marked diversity among breast tumors

**Fig. 4:**
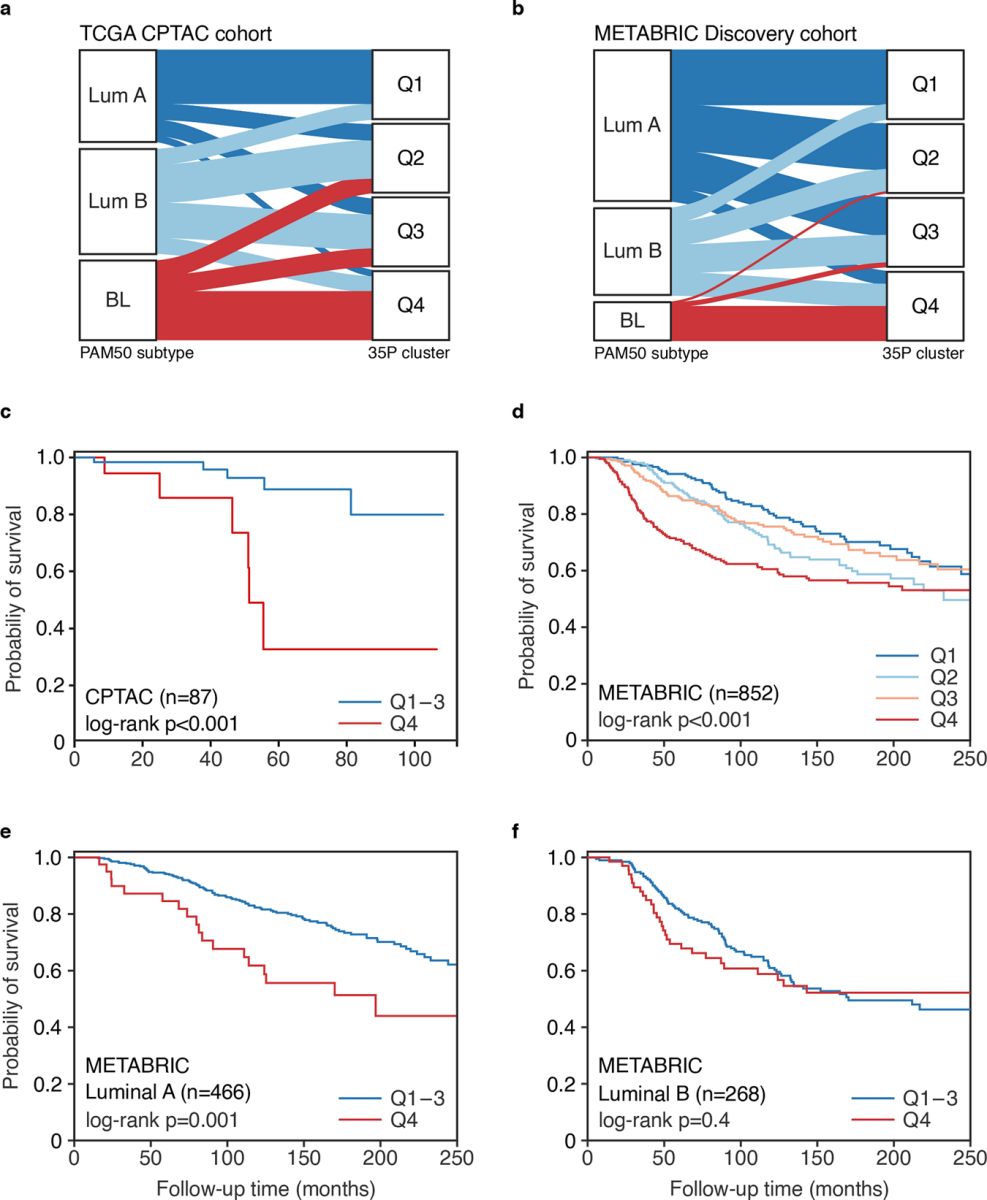
Survival analysis of CPTAC-TCGA^8^ and METABRIC-Discovery^12^ cohorts stratified by 35P stromal signature. **a**, **b** Distribution of 35P scores (quartiles) across PAM50 subtypes in TCGA CPTAC and METABRIC. **c** High 35P signature score (Q4) predicted poorer survival in the CPTAC-TCGA cohort (log-rank <0.001; overall survival; luminal-like and basal-like patient samples included, *n* = 87). **d**–**f** High 35P score was associated with worse breast cancer-specific survival in the METABRIC Discovery cohort (*p* < 0.001; all patients, *n* = 852), and luminal A subtype (*p* = 0.001; *n* = 466; Q4 *n* = 40; Q1–3 *n* = 426), but not in luminal B (*n* = 268; Q4 *n* = 69; Q1–Q3 *n* = 199). *p* values were calculated using the log-rank test. CPTAC Clinical Proteomic Tumor Analysis Consortium, METABRIC Molecular Taxonomy of Breast Cancer International Consortium, TCGA The Cancer Genome Atlas.

To explore the stromal proteome diversity among breast tumors based on expression of 35P proteins, we used the TCGA CPTAC^8^ (proteomic data; *n* = 87; HER2-subgroup excluded) and the METABRIC Discovery cohort^12^ (microarray data; mRNA values; *n* = 852; HER2 and normal-like excluded). Each patient-identifier was ranked from low to high 35P-score and grouped into quartiles (Q1–Q4). Notably, for both datasets, a marked diversity between epithelial-based tumor subtypes and the level of stromal 35P was observed. Both luminal A and luminal B cases were represented in all four quartiles of 35P (Q1–Q4), whereas basal-like cases were linked to three quartiles (Q2–Q4) (Fig. 4a, b). These findings indicate that there is no simple relationship between the epithelial and stromal proteome patterns in breast cancer.

We then compared the expression differences between the upper and lower quartiles (Q4 versus Q1) of 35P, overall, and separately for each tumor subtype. The most striking results were found within the luminal A subtype, where we found by GSEA that the Q4 subgroup was significantly enriched in features associated with “high-grade” tumors, such as hypoxia, inflammatory response, angiogenesis, and epithelial-to-mesenchymal transition (all FDR < 0.05, Supplementary Table 4). Notably, established signatures representing these processes correlated significantly with 35P (Supplementary Fig. 7). The data suggest that even within low-grade luminal A cases, aggressive tumor features were reflected in the stromal proteome.

Next, we investigated all differentially expressed genes between Q4 and Q1 of 35P in the luminal A subgroup (FDR < 0.05, FC > 1.5; 287 genes; Supplementary Table 5), with special emphasis on markers used for breast cancer classification. Estrogen receptor (ESR1) was downregulated in the Q4 group (FC = 0.5). Notably, none of the commonly used basal and luminal cytokeratin markers (CK5, CK 14, CK8/18) were significantly different between the two groups. The most up- and down regulated genes were MMP9 and PIP, respectively, both of which are linked to breast cancer invasion^15–18^, supporting that 35P reflects stroma-related biology.

By network analysis, we found that differentially expressed genes between Q4 and Q1 of 35P reflect a highly connected set of proteins (PPI enrichment *p* value < 1.0 × 10^−16^; Supplementary Fig. 8). This network contained three sub-networks consisting of genes with higher expression in Q4 compared with Q1. The proteins with the highest fold change in each subcluster were CXCL9 and CXCL10 (subcluster-1), MMP9 and CXCR4 (subcluster-2) and GBP1 and EPSTI1 (subcluster-3). In the breast, CXCL9, CXCL10, MMP9 and EPSTI1 show highest expression in macrophages (ProteinAtlas^19^). CXCR4 is most frequently associated with T-cells, and GBP1 with adipocytes and endothelial cells. These data support that proteins from TME-based cells are linked to 35P and associated with breast cancer aggressiveness.

### The 35P panel adds clinical and prognostic information beyond epithelial-based tumor subtypes

We hypothesized that the 35P stromal proteome signature might be used to improve stratification of breast cancer, based on clinical correlates and prognostic impact. To test the hypothesis of differences in patient survival, we first looked at 35P in the TCGA proteomics dataset analyzed by the Clinical Proteomic Tumor Analysis Consortium (CPTAC)^8^. In this cohort of 87 patients (luminal-like and basal-like), 31 of the 35P signature proteins were identified. Each patient-identifier was ranked from low to high 35P-score and grouped into quartiles (Q1–Q4). We found a significantly lower overall survival in the 35P high (Q4) group compared to 35P low (Q1–Q3; log-rank = 0.001; Fig. 4b).

Next, to further validate 35P in a separate dataset with significant follow-up, we applied the METABRIC Discovery cohort (mRNA values; *n* = 852; HER2 and normal-like excluded)^12^. Given that extracellular matrix proteins correlate poorly, and sometimes negatively, with mRNA expression, as reported in the OSLO2-study by Johansson et al.^7^, we first examined the mRNA-protein correlations for 35P. Of the 28 proteins that could be matched with protein-mRNA correlation data, 18 (64%) showed significant positive correlation with mRNA expression, and none showed significant negative correlation (Supplementary Table 6). This suggested that mRNA expression levels corresponding to the 35P signature reflect the protein phenotype. Overall, we found significantly lower probability of breast cancer specific survival in the 35P-high group (cut-off upper quartile, Q4; basal-like and luminal-like subtypes included; log-rank test, *p* < 0.001; Fig. 4d). We then stratified for subtype and found significantly lower probability of breast cancer specific survival for the 35P-high subgroup within luminal A tumors (*p* = 0.001; Fig. 4e), although not in luminal B (*p* = 0.46; Fig. 4f) or basal-like subtypes (*p* = 0.92). Notably, 35P was not associated with survival in the HER2-subgroup (*n* = 87). High 35P-score was associated with lower probability of recurrence-free survival among all cases (log-rank test, *p* < 0.001) and patients with luminal A breast cancer (log-rank test, *p* = 0.005) in the online KMplotter cohort (*n* = 2032)^20^ (Supplementary Fig. 9), where luminal B showed a borderline significance (*p* = 0.052) and basal-like was significant (*p* = 0.024).

In the METABRIC Discovery cohort^12^, 35P predicted lower breast cancer specific survival independent of basic prognostic factors tumor diameter, histologic grade and lymph node status, as well as the PAM50-based molecular subtypes (Cox’ regression, Wald test, *p* = 0.009, Table 1). When stratifying the cohort into luminal A, luminal B, and basal-like subtypes, the signature was still significantly independent of basic prognostic factors within the luminal A subtype (*p* = 0.002) (Supplementary Table 7). 35P remained significant within the luminal A-subtype even after adjusting for other stromal and immune-related signatures^21–25^.

**Table 1:** Multivariate survival analysis (breast cancer deaths; proportional hazards regression model) of breast cancer patients (METABRIC Discovery cohort; *n* = 852; luminal-like and basal-like cases included)

Variable	*n*	Univariate analysis		Multivariate analysis^a^	
		HR (95% CI)	*p* value^b^	HR (95% CI)	*p* value^b^
All patients (*n* = 852)^c^					
Tumor size					
<20 mm	263	1.00	<0.001	1.00	**0.001**
≥20 mm	589	1.90(1.44–2.52)		1.60(1.12–2.14)	
Histologic grade					
1–2	443	1.00	<0.001	1.00	**0.003**
3	409	1.83(1.44–2.32)		1.48(1.14–1.92)	
Lymph node status					
Negative	453	1.00	<0.001	1.00	**<0.001**
Positive	399	2.04(1.61–2.59)		1.74(1.36–2.22)	
PAM50 subtype					
Luminal-like^d^	734	1.00	0.02	1.00	0.278
Basal-like	118	1.45(1.06–1.98)		0.80(0.54–1.19)	
35P Stromal panel					
Q1–Q3	639	1.00	<0.001	1.00	**0.009**
Q4	213	1.67(1.29–2.13)		1.53(1.11–2.09)	
Luminal A (*n* = 454)					
Tumor size					
<20 mm	162	1.00	<0.001	1.00	**0.002**
>20 mm	292	2.20(1.42–3.42)		2.04(1.30–3.18)	
Histologic grade					
1–2	329	1.00	0.012	1.00	0.079
3	125	1.64(1.12–2.42)		1.42(0.96–2.10)	
Lymph node status					
Negative	266	1.00	0.010	1.00	**0.041**
Positive	188	1.63(1.12–2.37)		1.48(1.02–2.16)	
35P Stromal panel					
Q1–Q3	418	1.00	0.006	1.00	**0.009**
Q4	36	2.14(1.24–3.69)		2.09(1.21–3.63)	
Luminal B (*n* = 250)					
Tumor size					
<20 mm	62	1.00	0.005	1.00	0.086
>20 mm	188	2.03(1.24–3.30)		1.58(0.94–2.66)	
Histologic grade					
1–2	98	1.00	0.081	1.00	0.42
3	152	1.43(0.96–2.13)		1.19(0.78–1.79)	
Lymph node status					
Negative	121	1.00	<0.001	1.00	**<0.001**
Positive	129	2.38(1.60–3.53)		2.07(1.37–3.13)	
35P Stromal panel					
Q1–Q3	187	1.00	0.79	1.00	0.72
Q4	63	1.06(0.69–1.64)		1.09(0.69–1.71)	

*CI* confidence interval, *aHR* adjusted hazard ratio, *n* number of patients, *NS* not significant.

^a^All confounders were included in the table: tumor size, histologic grade, lymph node status, PAM50 and 35P. PAM50 was not included when stratifying patients into luminal A or luminal B subtypes.

^b^*p* values correspond to the hazard ratio for each covariate in the model. *p* < 0.05 in the mulivariate analysis is shown in bold.

^c^Only patients with luminal A, luminal B and basal-like breast cancers were included (*n* = 852, METABRIC discovery cohort^55^).

^d^Luminal-like: patients with luminal A and luminal B breast cancer subtypes.

35P was independent of the basic prognostic factors. Still, we wanted to investigate if 35P also had prognostic value in the small group of patients with the most advanced tumors, and we used the Nottingham Prognostic Index (NPI)^26^ to identify patients with poor prognosis (NPI ≥ 5.4). Overall, 35P was significantly associated with breast cancer-specific survival in the NPI low ( <5.4) group, but not in the NPI high ( ≥5.4) group (Supplementary Fig. 10a, b; METABRIC cohort). We then looked at 5-year survival and found a significant association with 35P within the NPI-high group, indicating a time-dependent relationship (*p* = 0.01; data now shown). In patients with luminal A breast cancer, high 35P is associated with poorer survival in both NPI-low and NPI-high groups (Supplementary Fig. 10c, d), supporting the independent nature of 35P.

Next, we examined 35P-associations with other relevant variables such as tumor cellularity, the 10 integrative clusters (IntClust) from the METABRIC study^12^ and hormone therapy. Two subgroups of ‘tumor cellularity’ are included in the METABRIC cohort (moderate/high cellularity). Cases with high cellularity showed a significant difference for 35P (by upper quartile), while those with moderate cellularity did not, and there was no significant interaction with cellularity in multivariate models (all patients or luminal A cases only). Then, we examined the relationship between 35P and IntClust subgroups^12^. Interestingly, both remained significant in multivariate analysis, indicating that they capture different features of tumor biology (Supplementary Table 8). Luminal A cases with high 35P-score were distributed across many IntClust subgroups, supporting independent value of 35P (Supplementary Fig. 11). We also stratified for hormone therapy and found that high 35P score was associated with worse outcome in both treated and untreated patients (Supplementary Fig. 12). By multivariate analysis, 35P was significant, whereas therapy was not, and there was no interaction between the two.

**Fig. 5:**
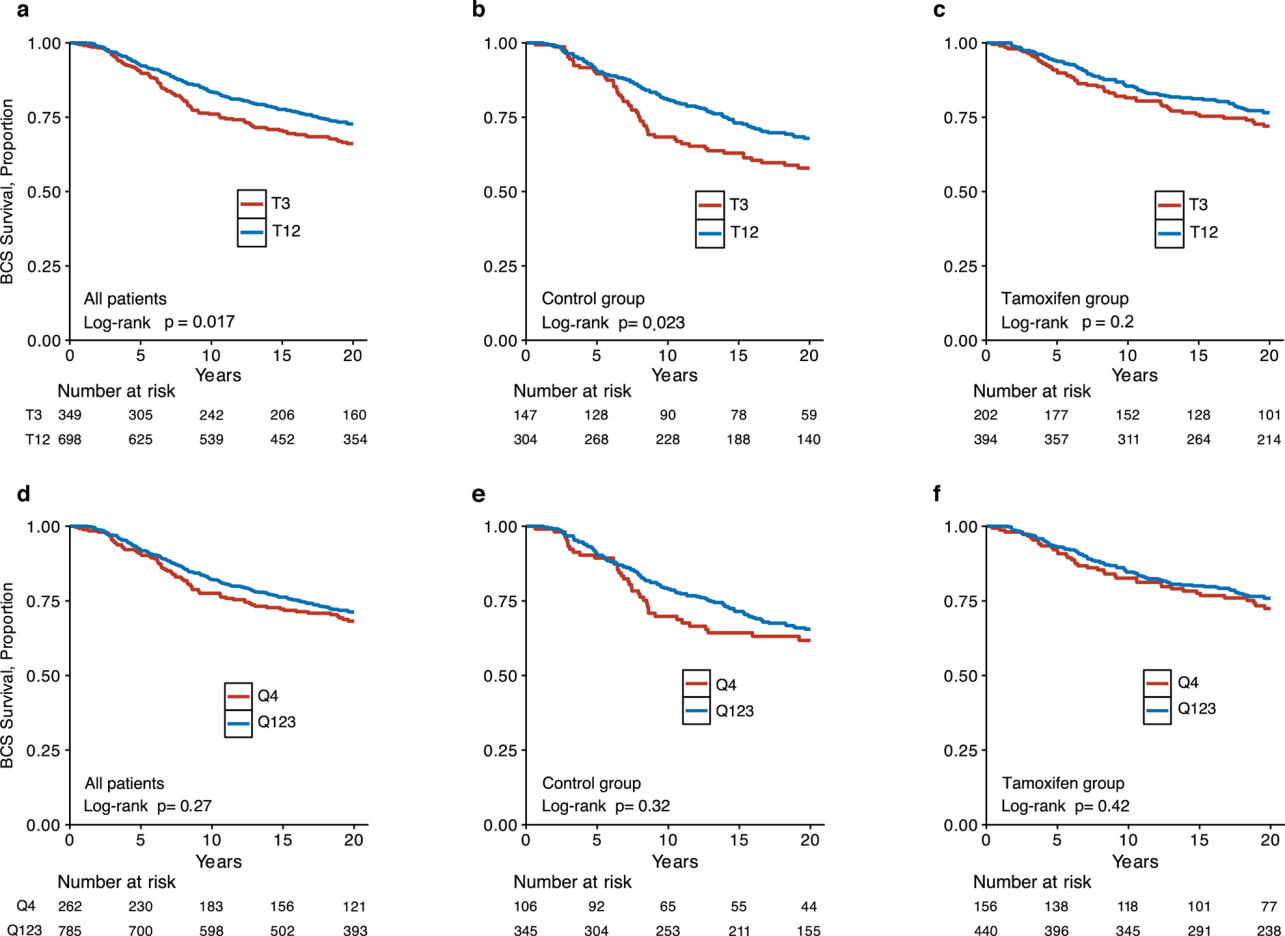
Univariate long-term survival of ER+/HER2‒ breast cancer patients from the STO trials. Two cutoffs are presented with Breast Cancer-Specific Survival (BCSS) of all patients (*n* = 1047), control group (*n* = 451) and tamoxifen group (*n* = 596). **a**–**c** Tertile cutoff at the upper tertile (T3), and **d**–**f** quartile cutoff at the upper quartile (Q4) versus the rest.

We then asked whether the 35P stromal proteome signature could predict patient survival in the setting of a large randomized clinical trial (RCT), the STO-trials. In the STO-trials^27^, patients with ER-positive/HER2-negative primary tumors (*n* = 1047) were included in the analyses. Two cutoffs were analyzed, one more conservative using the upper quartile (Q1–Q3 vs. Q4), similar to what was done for the other cohorts, in addition to using the upper tertile (T1–T2 vs. T3) as a cutoff. In the analyses using the upper tertile cutoff, a high 35P level (T1–T2 vs. T3) was significantly associated with poorer long-term (20-year) distant recurrence free-interval (DRFI) and breast cancer specific survival (BCSS) for ER+/HER2− patients (DRFI and BCSS: log-rank *p* = 0.029 and *p* = 0.017, respectively) and in the control arm (DRFI and BCSS: log-rank *p* = 0.033 and *p* = 0.023, respectively) (Fig. 5 and Supplementary Fig. 13). No significant difference between high and low (T3 vs. T1–T2) 35P levels was observed in the tamoxifen treatment arm. However, by multivariate analysis, high (T3 vs. T1–T2) 35P-score was significantly associated with increased risk of BCSS in the tamoxifen group (*n* = 566; HR 1.59, 95% CI: 1.06–2.38), but not in the control arm (Table 2). When stratifying for PAM50 luminal A and luminal B in the multivariate analysis, we observed an association between high (T3 vs. T1–T2) 35P and worse survival in patients with luminal B breast cancer in the tamoxifen group (*n* = 147; HR 2.61, 95% CI: 1.29–5.30). There was no significant statistical interaction between 35P expression level and tamoxifen treatment. Further, using the quartile cutoff resulted in a lower sample size, especially for the subgroup analyses. No significant associations between the 35P expression level high versus low (Q4 vs. Q1–Q3) were observed (Fig. 5, Supplementary Fig. 13 and Supplementary Table 9).

**Table 2:** Multivariate survival analysis of 35P score and risk of breast cancer-specific deaths in patients from the STO trials^27^

	35 P score	Patients	BCD^c^ 20 yr	aHR^b^ (95% CI)	35 P score	Patients	BCD^c^ 20 yr	aHR^b^ (95% CI)
	Tertile cutoff^a^	No.	No.	BCSS^c^	Quartile cutoff^a^	No.	No.	BCSS^c,d^
ER+HER2− patients	T1–T2	662	167	1.00 (ref.)	Q1–Q3	743	199	1.00 (ref.)
	T3	332	103	1.27 (0.97–1.65)	Q4	251	71	1.15 (0.86–1.55)
Control	T1–T2	285	85	1.00 (ref.)	Q1–Q3	326	105	1.00 (ref.)
	T3	143	54	1.24 (0.86–1.79)	Q4	102	34	1.11 (0.73–1.68)
Tamoxifen	T1–T2	377	82	1.00 (ref.)	Q1–Q3	417	94	1.00 (ref.)
	T3	189	49	**1.59 (1.06–2.38)**	Q4	149	37	1.38 (0.89–2.14)
Luminal A patients	T1–T2	437	90	1.00 (ref.)	Q1–Q3	490	104	1.00 (ref.)
	T3	214	46	1.29 (0.88–1.91)	Q4	161	32	1.21 (0.79–1.86)
Control	T12	194	49	1.00 (ref.)	Q1–Q3	218	59	1.00 (ref.)
	T3	91	26	1.51 (0.88–2.60)	Q4	67	16	1.21 (0.65–2.24)
Tamoxifen	T1–T2	243	41	1.00 (ref.)	Q1–Q3	272	45	1.00 (ref.)
	T3	123	20	1.10 (0.60–2.01)	Q4	94	16	1.28 (0.67–2.42)
Luminal B patients	T1–T2	157	61	1.00 (ref.)	Q1–Q3	179	77	1.00 (ref.)
	T3	97	48	1.24 (0.80–1.94)	Q4	75	32	0.98 (0.62–1.57)
Control	T1–T2	63	28	1.00 (ref.)	Q1–Q3	77	37	1.00 (ref.)
	T3	44	25	0.94 (0.47–1.90)	Q4	30	16	1.07 (0.53–2.16)
Tamoxifen	T1–T2	94	33	1.00 (ref.)	Q1–Q3	102	40	1.00 (ref.)
	T3	53	23	**2.61 (1.29–5.30)**	Q4	45	16	1.45 (0.66–3.18)

^a^Two cutoffs are presented. Tertile cutoff at the upper tertile (T3) and quartile cutoff at the upper quartile (Q4) versus the rest. Patients with unknown values (*n* = 53) for tumour size, tumour grade, Ki67 status, and PR status were excluded prior to Cox regression analyses.

^b^aHR: adjusted hazard ratio. Multivariable Cox proportional hazard regression adjusted for age, menopausal status, random assignment period, tumor size, tumor grade, lymph node status, PR status, Ki67-status, type of surgery, chemotherapy, and radiotherapy status. aHR with 95% confidence intervals that do not cross 1 are shown in bold.

In addition: models in the overall ER+/HER2− cohort included tamoxifen therapy and PAM50 subtype; ER+/HER2− models stratified by treatment (control vs. tamoxifen) included PAM50 subtype but not tamoxifen therapy; models in the overall Luminal A/Luminal B cohorts included tamoxifen therapy but not PAM50 subtype; models restricted to tamoxifen-treated Luminal A/Luminal B patients did not additionally adjust for tamoxifen or PAM50 subtype.

^c^BCD breast cancer deaths, BCSS breast cancer-specific survival.

^d^There were no significant statistical interactions with treatment.

Taken together, the 35P-high group showed a worse outcome, which indicates that stromal proteomic information may be necessary to classify breast cancer as precisely as possible. As an independent prognostic factor, 35P might be relevant in both luminal A and luminal B cases, dependent on the context. Notably, the fact that 35P was independently significant when based on mRNA values from whole tissue samples supports the robustness of the 35P signature.

### Reduction analysis of the 35P stromal panel shows preserved significance

To investigate whether the number of proteins in 35P could be reduced while preserving its prognostic power, we iteratively removed one protein and tested the predictive power of each 34-protein subsets in the METABRIC Discovery cohort (all patients; *n* = 852; upper quartile; log-rank test)^12^. The subset with the best predictive power (i.e., lowest log-rank *p* value) was retained, and the process was repeated until only one protein remained (Supplementary Fig. 14). A subset of 18 proteins showed the strongest prognostic power, and a subset of only 5 proteins also had strong prognostic prediction. For both of these subsets, a high score was significantly associated with poor prognosis in patients with luminal A breast cancer. These data suggest that 35P can be reduced to smaller subsets, which may be of clinical interest.

## Discussion

In this study, we aimed to explore the global proteome of the breast cancer microenvironment, and we wanted to examine whether stromal-based information could improve patient stratification beyond current tumor cell-based classification. By laser capture microdissection and separation of the stromal and epithelial tumor compartments combined with MS-based proteomics, we found that a stromal protein panel (35P) appears to improve prognostic separation. This indicates that breast cancer classification should possibly incorporate TME-based information, in addition to tumor cell characteristics.

Our findings revealed that the epithelial proteome clustered in accordance with current breast cancer classification (basal-like versus luminal-like). In contrast, the stromal proteome was different, demonstrating three subgroups across epithelial-based tumor categories by unsupervised clustering. This indicates that additional information might be extracted from the TME compartment as reflected in the stromal tissue. We here discovered a 35-protein (35P) stromal-based panel that could separate patients based on survival in several independent patient cohorts, including a large randomized clinical trial of hormone receptor-positive and HER2-negative breast cancer with long-term follow up (20 years)^27^.

The 35P panel showed independent prognostic influence by multivariate survival analysis, in addition to standard molecular subtypes and basic pathology factors. Notably, our data indicate that 35P, as an independent factor, might be relevant in both luminal A and luminal B tumor categories, dependent on the tumor context and with different cutoff values for 35P. In the METABRIC cohort, which includes larger and more aggressive tumors than the STO trial, 35P was most relevant in the luminal A category, as it was in the KMplotter series. In the large STO cohort with long-term follow-up, 35P appeared to be relevant in the luminal B subgroup and in the treatment arm, where additional factors have been adjusted for, including other treatments^27^. Taken together, these data might support a potential clinical value of TME-based information across luminal subcategories.

The epithelial and stromal tumor compartments do not always parallel each other^28,29^, as supported by our present observations. Notably, luminal A patients were represented in all four quartiles (Q1–Q4) of the 35P-score, demonstrating stromal diversity at the proteome level, even among luminal A tumors. Discordant luminal A cases, assumed to be low-grade with an excellent prognosis, might still develop a more “high-grade” stroma and show worse outcome than expected, as revealed by our findings. It is well known that some luminal A tumors have a worse prognosis than the rest^30,31^, and our data indicate that the stromal proteome panel (35P) might identify such cases with independent value.

Functional deconvolution of the 35P stromal panel indicates a link to ECM biology, pointing to key processes for tumor progression such as hypoxia, angiogenesis, and EMT. In addition, matched proteome and single-cell imaging mass cytometry (IMC) data indicated that 35P-high cases are linked to higher abundance of tumor-associated macrophages (TAM). This finding was validated for luminal A tumors using both for gene expression data and single-cell IMC data from the large METABRIC Discovery cohort^12,13^, and our results support the view of macrophages as important in the breast cancer microenvironment^32,33^.

As part of 35P validation, we included mRNA data from the METABRIC Discovery cohort^12^ and the STO-trials^27^. This may present as a limitation, as mRNA and protein expression show variable correlation depending on, e.g., location and function. However, as most of the 35 P proteins showed significant positive protein-mRNA correlations^7^, we consider it appropriate to use these mRNA values as proxies for protein expression. Importantly, 35P demonstrated a significant impact on disease progress when using corresponding mRNA data derived from whole tissue samples. This supports the robustness of 35P, and it also indicates that regular tissues could be applied in a routine setting, without the microdissection step used in our discovery phase. However, we cannot rule out that bulk tissue and mRNA data have underestimated the impact of 35P, underscoring the need for large-scale proteomic breast cancer datasets with long-term follow-up. Of further interest for potential practical applications, our signature reduction analysis indicates that a subset of 18 proteins, and possibly down to 5 proteins, may be used instead of the full 35P panel. This needs to be further studied.

In summary, this study of compartment-specific breast cancer proteomes indicates that stromal information can improve tumor classification beyond current criteria, with relevance in both luminal A and luminal B cases. The stromal panel (35P-high), indicating a ‘high-grade stroma’, likely reflects a more permissive microenvironment, and information from the TME may provide better patient stratification with potentially improved treatment, by identification of more aggressive luminal tumors.

## Methods

### Bergen Breast Cancer Cohort (BBCC)

Breast cancer tissue samples (FFPE) were collected from patients (aged 50–69 years) diagnosed with invasive breast carcinoma during 1996–2003 as part of the prospective and population-based Norwegian Breast Cancer Screening Program (*n* = 534)^30^. Primary tumors with a diameter larger than 5 mm were randomly selected, with a balance between luminal-like and basal-like tumors. Initially, 50 cases were included for whole tissue proteomics; four cases had insufficient remaining tissue, and 46 cases were finally included. Subsequently, 24 cases (12 basal-like, 6 luminal A, 6 luminal B) were selected for laser capture microdissection (LCM), with separation of epithelial and stromal tissue compartments. Among these, all basal-like tumors were triple-negative, and all luminal-like samples were estrogen and progesterone receptor positive and HER2-negative. Luminal B tumors displayed more than 14% Ki67-positive nuclei^34^, according to national guidelines at the time. All cases were treatment naïve at the time of surgery. To meet these criteria, 22 of 24 cases were randomly selected from the complete BBCC cohort and overlapping with the whole-tissue proteomics cases. In addition, two cases (one luminal A, one luminal B) were added from the complete cohort. Luminal A, luminal B, and basal-like classifications were assigned by experienced breast pathologists using immunohistochemical (IHC) proxy criteria prior to laser capture microdissection and discovery studies by proteomic analyses.

### External datasets

To explore protein and gene expression patterns corresponding to the stromal protein signature found in breast cancer, we mapped the signature to publicly available proteomic and mRNA datasets with annotations on survival, clinico-pathological data, and molecular tumor subtypes defined by the PAM50 algorithm^35^ (TCGA-CPTAC^8^, *n* = 87; HER2+ cases excluded; METABRIC Discovery cohort^12^, *n* = 852; HER2 and normal-like subtypes were excluded; and the KMplotter database (*n* = 2032)^20^).

To further validate the clinical impact of the 35P signature, we analyzed ER+/HER2− breast cancer patients (*n* = 1,047) from the randomized Stockholm Tamoxifen Trials (STO). Luminal A (*n* = 688) and luminal B (*n* = 264) subtypes were defined using PAM50^27,36^. Microarray gene expression profiles from bulk breast cancer tissues (macrodissected tumor tissue to ensure ≥*30%* tumor cells) were available for all patients. The STO-trials enrolled women with invasive breast cancer (1976–1996) using population-based inclusion. Patients were randomized to at least 2 years (2 or 5 years) of adjuvant tamoxifen therapy (40 mg daily) versus no endocrine therapy (control). Twenty-year follow-up until December 31, 2016, was available for all patients from Swedish high-quality national and regional registries of high validity and essentially complete coverage^37–39^.

The METABRIC Discovery cohort (used here) and the STO cohort were compared for basic clinico-pathologic factors. As expected, METABRIC cases are older, with larger tumors, higher histologic grade and more lymph node metastases (all highly significant), from cross-table comparisons (not shown), and with shorter follow-up compared with the STO cohort. This is relevant for the interpretation of our findings and the discussion of slightly different cutpoints applied.

We used publicly available imaging mass cytometry (IMC) data from Danenberg et al.^13^, which analyzed tumor samples from the METABRIC cohort.

### Tissue macrodissection and microdissection

Whole tissue samples were macro-dissected to ensure ≥*30%* tumor cells using a scalpel and a matching HE slide for guidance to enrich for tumor areas. For microdissection, 10 µm thick FFPE sections were deparaffinized, rehydrated and stained with hematoxylin. Tumor epithelium and stromal compartments were laser microdissected (PALM MicroBeam, Zeiss) and pressure catapulted into tube caps (AdhesiveCap 500 opaque, Zeiss). Depending on the available tissue, 0.5–1.9 × 10^7^ µm^3^ were microdissected. When dissecting tumor cells, we aimed to select representative areas from the leading edge (or tumor periphery) where possible, and to avoid areas with necrosis or scarring. We also aimed to dissect tumor-cell adjacent stromal areas (see Supplementary Fig. 15 for examples of laser microdissected areas). To assess cross-contamination of tumor cells in the stromal compartment, we compared protein intensities of the epithelial marker cytokeratin-8, as previously performed on the same material^40^. In short, very low levels of tumor cell contamination (mean 1.6%; median 1.7%) were found in the stromal compartments.

### Sample preparation

The samples were prepared using the FFPE-FASP protocol, which is described in detail elsewhere^41^. After enzymatic digestion of proteins, the resulting peptides were eluted from the FASP filters and desalted using Oasis HLB µElution plates (Waters, Milford, MA, USA).

### Mass spectrometry analysis

The samples were analyzed in their entirety during a 180 min reverse-phase gradient on a Q-Exactive HF mass spectrometer (Thermo Fisher Scientific, Waltham, MA, USA) connected to a Dionex Ultimate NCR.3500RS LC system. Samples were dissolved in a 2% acetonitrile (ACN)/0.1% formic acid (FA) and trapped in the pre-column (Dionex, Acclaim PepMap 100, 2 cm × 75 µm i.d., 3 µm C18 beads) in loading buffer (0.1% trifluoroacetic acid), at a flow rate of 5 µL/min for 5 min, before separation by reverse phase chromatography (PepMap RSLC, 25 cm × 75 µm i.d., EASY-spray column, packed with 2 µm C18 beads) at a flow rate of 200 nL/min. Solvent A and B were 0.1% FA (vol/vol) in water and 100% ACN, respectively. The gradient composition was 5% B from 0 to 5 min, which increased linearly to 8% B from 5 to 5.5 min to 24% B from 5.5 to 115 min, to 35% B from 115 to 140 min, and to 90% B from 140 to 155 min. Washing and conditioning of the column were performed from 155 to 170 min with 90% B, and reduced to 5% B from 170 to 180 min. The MS instrument was equipped with an EASY-spray ion source (Thermo Fisher Scientific, Waltham, MA, USA) and was operated in data-dependent-acquisition mode. Instrument control was performed using Q-Exactive HF Tune 2.4 and Xcalibur 3.0. MS spectra were acquired in the scan range 375–1500 m/z with resolution *R* = 120,000 at *m*/*z* 200, with an automatic gain control (AGC) target of 3.0 × 106 and a maximum injection time (IT) of 100 ms. The 12 most intense eluting peptides above intensity threshold 5.0 × 104, with charge states 2 or larger, were sequentially isolated to a target AGC value of 1.0 × 105, with resolution *R* = 30,000, an IT of 110 ms and a normalized collision energy of 28%. The isolation window was set to 1.6 m/z with an isolation offset of 0.3 and a dynamic exclusion of 25 s. Lock-mass internal calibration was used.

### Processing raw mass spectrometry data

Raw mass spectrometry (MS) data were processed using MaxQuant (v1.6.0.16)^42^, using recommended settings for label-free quantification^43^. Identified features were cross-checked against the “reference proteome” database from UniProt.org (full proteome analysis; FASTA file downloaded October 2017) or the core matrisome database^10^ (ECM protein analysis). The processed MS data were analyzed with Perseus (v1.6.0.7)^44^. Protein intensities were log_2_-transformed and grouped according to breast cancer subtype. Identified proteins were filtered to retain only proteins with intensity data (valid values) in at least 50% of samples within one group.

### Protein expression correlation analysis

Protein intensity values were correlated between samples, and the correlation coefficients (*r*_*s*_) were visualized by unsupervised hierarchical clustering (distance: Euclidean; linkage: average).

### Imaging mass cytometry (IMC)

We used a 36-marker antibody panel to map the spatial landscape of the 24 laser microdissected cases from the Bergen Breast Cancer Cohort (BBCC). The overall methods are reported in Bjørnstad et al.^45^, with some modifications. In short, cell segmentation was performed with DeepCell/Mesmer using the Steinbock pipeline^46^. Spill-over correction and batch-effect correction (Harmony) were performed by following the “Analysis workflow for IMC data” (https://bodenmillergroup.github.io/IMCDataAnalysis/index.html)^46^. The markers used to identify immune cells in the current study were: CD3 (T-cells), CD4 (CD4+ T-cell), CD8 (CD8+ T-cell), CD20 (B-cell), CD45 (pan-immune), CD68 (M1-macrophage), and CD163 (M2-macrophage). All antibodies were validated on IHC and IMC using a tissue microarray with positive control tissue for each marker. The IMC antibody panel is presented in Supplementary Table 10. The IMC dataset is available via the Zenodo data repository (10.5281/zenodo.18254639).

### Immune infiltration scoring

Two methods were used to investigate immune cell infiltration for each case: (1) manual counts from H&E-whole sections, and (2) estimation by imaging mass cytometry (IMC on TMA cores), spatial single-cell data for immune cell content.

#### Manual estimation of immune cells

Parallel sections to those used for microdissection were stained with H&E, and immune cell content in the areas that were microdissected was manually assessed in a blinded manner using a semi-quantitative scale from 1 to 4: 1 = none/very low; 2 = low (less than 10% of stromal area); 3 = moderate (less than 50% of area); 4 = high (more than 50% of area).

#### Imaging mass cytometry-based immune cell estimation

In-house IMC data from the 24 cases included for laser microdissection were assessed to evaluate the immune cell content for each case. Specifically, the numbers of T-cells, B-cells, and macrophages were counted, and any discrepancies in scanned area were adjusted for. For both scoring methods, differences between groups were evaluated using the Mann–Whitney *U*-test.

### Protein and gene expression signature scoring

Each protein/gene signature was zero-mean normalized by subtracting the average expression value (across all patient samples) from the expression value of each patient sample. The signature score was the sum of normalized expression values from upregulated proteins minus the sum from downregulated proteins.

### Gene ontology (GO) analysis

GO analyses were performed using PANTHER Classification System^47^ (PANTHER 16.0, Overrepresentation Test, GO Ontology database DOI: 10.5281/zenodo.4735677 Released 2021-05-01).

### Gene deconvolution

Gene expression data were deconvoluted using the Microenvironment Cell Populations-counter^14^ (MCP-counter, v1.2.0) package implemented in R (v4.4.0), following the instructions provided at https://github.com/ebecht/MCPcounter. The method estimates the relative abundance of pre-defined immune and stromal cell populations based on transcriptomic markers and was used to compare cell populations between groups.

### Gene sets enrichment analysis (GSEA)

GSEA was used to identify enriched Hallmark gene sets (MSigDB)^48^ between the Q4 and Q1 groups, using Qlucore Omics Explorer 3.7 (Qlucore AB, Lund, Sweden). The genes were ranked using a two-sided Student’s *t* test.

### Network analysis

The protein-protein interaction network was generated using StringDB^49,50^ (v11.5) and Cytoscape software^51^ (v3.8.2) and the core app NetworkAnalyzer^52^ (v4.4.6) for computing basic network properties. For identifying subclusters of proteins, the Cytoscape add-on MCODE^53^ (v2.0.0) was used with the following settings: include loops: false; degree cutoff: 2; haircut: true; fluff: false; node score cutoff: 0.2; K-Core: 2; max. depth from seed: 100.

### Signature reduction analysis

From a parent set of proteins (*n*), one protein was iteratively removed, and each combination of the resulting subset (*n*−1) is evaluated by an appropriate statistical test. The best performing protein subset is retained (here: lowest *p* value, log-rank test). This process was repeated until a stop criterion was reached (here: only one protein remained).

### Statistics

Fisher’s exact test was used to test categorical clinical variables, and the Mann–Whitney *U*-test was used to test continuous clinical variables. Differential abundance of proteins was tested using Student’s *t* test and multiple-sample ANOVA test. Spearman’s rank correlation was used to test nonparametric correlations.

The TCGA-CPTAC^8^ and METABRIC^12^ data were analyzed using SPSS (Statistical Package of Social Sciences), Version 25.0 (Armonk, NY, USA; IBM, Corp). Alluvial plots were generated using the *alluvial* R-package (v.0.1-2; https://github.com/mbojan/alluvial). Non-parametric bivariate correlations of continuous variables were tested by Spearman’s rank correlation test. For survival analyses, the endpoint was overall survival (TCGA-CPTAC), and breast cancer-specific survival (METABRIC), and recurrence-free survival (KMPlotter). Follow-up time was defined as the time from the date of diagnosis to the date of death or last follow-up.

Univariate survival analysis by the Kaplan–Meier method was performed using the log-rank test. Patients who died of other causes (METABRIC only) or who were alive at the last date of follow-up were censored. The influence of covariates on breast cancer-specific survival was analyzed by Cox’ proportional hazards multivariate method and tested by the enter method. All variables were tested using log-minus-log plots to determine their ability to be incorporated in multivariate modeling. When categorizing continuous variables, cut-off points were based on median, tertile, or quartile values, also considering the distribution profile, the size of subgroups, and the number of events in survival analyses. In multivariate analyses, tumor diameter, histologic grade, lymph node status, PAM50-based tumor subtypes, and 35P were generally included.

For the STO-trials, the long-term (20-year) tamoxifen treatment benefit was assessed by univariate Kaplan-Meier and multivariable Cox proportional hazards regression analysis. The endpoints were Distant Recurrence-Free Interval (DRFI) and Breast Cancer-Specific Survival (BCSS), including distant metastasis or fatal breast cancer as events. The multivariable analyses were adjusted for standard clinical patient and tumor characteristics, including age at diagnosis and randomization period, menopausal status, tumor size, tumor grade, lymph node status, PR status, Ki-67 status, chemotherapy, radiotherapy, and type of surgery (mastectomy or breast-conserving surgery). The R survival and R survminer packages were used for the Kaplan–Meier and Cox proportional hazard regression analyses. Analyses were performed in R version 4.3.1. The *p* value significance threshold was set to 0.05.

#### Differential cell abundance analysis

Cell type abundance differences between groups (Danenberg et al. IMC data)^13^ were tested using edgeR^54^ with a negative binomial generalized linear model. The count data were normalized for library size using TMM normalization. A likelihood ratio test was performed with gene signature expression levels as the predictor.

### Ethics

The study protocol was approved by the Western Norway Regional Committees for Medical and Health Research Ethics, REC West (REK 2014/1984). The informed consent was waived by the REC West Committee based on national guidelines, as well as the age and size of the full cohort covered by the approval. However, the patients included were informed about the research project with the possibility to withdraw consent. In total, nine women withdrew consent. The STO-trials were approved by the Karolinska Institutet Regional Ethics Committee with the Stockholm Regional Cancer Center as the trial center. Informed consent was obtained from the patients before randomization. The trials were approved and initiated before the practice of trial registration in Sweden. All work was performed in accordance with the Declaration of Helsinki.

## Supplementary information

Supplementary Information

## Data Availability

Proteomics data (in-house) used in this study have been published previously by Kjølle et al.^40^, and have been deposited at the Proteomics Identifications Database (PRIDE) as PXD027012 and are publicly available. The in-house IMC dataset is available from the Zenodo data repository (10.5281/zenodo.18254639). The raw RNA microarray data from the STO randomized controlled trial are assigned a (10.5878/3vxa-3c28). Data access requests may be submitted to the Swedish National Data Service through the DOI link and will be reviewed by the STO Trialists Group. In addition, this paper analyzes existing, publicly available data: TCGA Breast Cancer Proteome, accessible at NIH Proteomic Data Commons as PDC000173; METABRIC Discovery cohort, accessible at the European Genome-Phenome Archive as EGAD00010000210. Any additional information required to reanalyze the data reported in this paper is available from the corresponding author upon reasonable request. The data underlying the STO-analyses cannot be shared publicly because of the privacy of individuals who participated in the study.
