# Supplementary material for: Stromal-based proteome data improve stratification of hormone receptor-positive breast cancer
Source: NPJ Breast Cancer. 2026 Apr 9;12:81. doi: 10.1038/s41523-026-00943-y (PMC13249943; doi:10.1038/s41523-026-00943-y)
Supplement: Supplementary file 1 — Supplementary Information [file 41523_2026_943_MOESM1_ESM.pdf]

Stromal-based proteome data improve stratification of hormone receptor-positive breast cancer (Finne et al.)

Supplementary Information

Supplementary Tables 1 – 10

Supplementary Figures 1 – 15

References

Supplementary Table 1. Patient characteristics at time of biopsy (cases included in the discovery phase using laser capture microdissection of tumor stroma, n=24)

Variable	Breast cancer subtype		p-value
	Luminal-like (n=12)	Basal-like (n=12)	
Age (years) [a]	59.9 (6.0)	58.3 (6.3)	0.41
Histologic grade			< 0.001[b]
1	8 (67 %)	0 (0 %)	
2	3 (25 %)	2 (17 %)	
3	1 (8 %)	10 (83 %)	
Tumor diameter (mm) [a]	15.7 (6.3)	21.9 (9.3)	0.10
Lymph node metastasis			1.00
Absent	4	4	
Present	8	8	
Estrogen receptor positive	12 (100%)	0	-
Progesterone receptor positive	12 (100%)	0	-
Her2 positive	0	0	-
Ki67 positive tumor nuclei (%)[a]	19.0 (13.4)	66.9 (17.0)	<0.001

[a] Continuous values provided as mean with standard deviation inside parentheses and groups compared using independent samples Mann-Whitney U-test

[b] Categorical values provided with percent inside parenthesis; groups compared using Fisher's exact test

Supplementary Table 2. 35P stromal signature proteins

UniProtID	Gene names	Basal-like vs luminal-like	
		Fold change	P-value
P14543	NID1	5.56	0.001
Q6PCB0	VWA1	3.36	0.050
Q92626	PXDN	2.67	0.046
P01861	IGHG4 [a]	2.52	0.002
P24844	MYL9	2.40	0.019
O15116	LSM1	2.12	0.013
P61165	TMEM258	2.03	0.021
Q9UBR2	CTSZ	2.03	0.038
O00468	AGRN	2.02	0.037
Q53FA7	TP53I3	1.99	0.041
P40261	NNMT	1.98	0.029
P01624	IGKV3D-15 [a]	1.95	0.000
P01893	HLA-H	1.82	0.050
Q9HD20	ATP13A1	1.81	0.022
Q9UKY7	CDV3	1.77	0.022
O95486	SEC24A	1.75	0.042
O00303	EIF3F	1.72	0.020
P62328	TMSB4X	1.69	0.042
Q9UK45	LSM7	1.67	0.048
P11387	TOP1	1.66	0.034
A0A0C4DH25	IGKV3D-20 [a]	1.60	0.019
O95967	EFEMP2	1.59	0.041
P16152	CBR1	1.58	0.021
P48735	IDH2	1.55	0.023
O43516	WIPF1	1.53	0.044
P43686	PSMC4	1.52	0.028
Q8IVL6	LEPREL2	-1.53	0.042
Q8TES7	FBF1	-1.57	0.035
Q99536	VAT1	-1.69	0.003
P08253	MMP2	-1.86	0.029
P19827	ITIH1	-1.95	0.029
Q6NZI2	PTRF	-2.01	0.042
Q15661	TPSAB1	-3.78	0.002
P23946	CMA1	-6.47	0.048
P12273	PIP	-13.5	0.015

[a] Protein do not have a corresponding gene in the METABRIC discovery cohort.

Supplementary Table 3. Overrepresented gene sets (Gene Ontology) in 35P signature proteins

GOID	Biological process/cellular component		p-value[a]	FDR [b]
GO:0062023	Collagen-containing extracellular matrix	CC	1.80E-09	3.70E-06
GO:0030312	External encapsulating structure	CC	2.90E-08	1.90E-05
GO:0031012	Extracellular matrix	CC	2.80E-08	2.80E-05
GO:0005615	Extracellular space	CC	3.60E-07	1.80E-04
GO:0045229	External encapsulating structure organization	BP	2.50E-07	1.30E-03
GO:0005576	Extracellular region	CC	3.90E-06	1.60E-03
GO:0043062	Extracellular structure organization	BP	2.40E-07	1.90E-03
GO:0030198	Extracellular matrix organization	BP	2.30E-07	3.70E-03
GO:0005604	Basement membrane	CC	2.50E-05	8.40E-03
GO:1990726	Lsm1-7-Pat1 complex	CC	5.80E-05	1.70E-02
GO:0031982	Vesicle	CC	7.80E-05	1.90E-02
GO:0065010	Extracellular membrane-bounded organelle	CC	1.20E-04	2.10E-02
GO:0043230	Extracellular organelle	CC	1.20E-04	2.20E-02
GO:1903561	Extracellular vesicle	CC	1.20E-04	2.40E-02
GO:0070062	Extracellular exosome	CC	1.10E-04	2.50E-02

PANTHER 16.0, PANTHER Overrepresentation Test (Released 2021-02-24), GO Ontology database DOI 10.5281/zenodo.4735677 (released 2021-05-01)
[a] Raw p-value, Fisher's exact test.
[b] Benjamini-Hochberg false discovery rate (FDR).
BP: biological process. CC: cellular component. GOID: Gene ontology term unique identifier.

Supplementary Table 4. Gene set enrichment analysis of 35P-high (Q4) versus 35P-low (Q1) among luminal A patients in the METABRIC Discovery cohort (FDR<0.05)

Hallmark gene set [a]	Matches	Enrichment score	p-value	FDR
Interferon gamma response	193 / 200	0.74	0.004	0.005
Complement	190 / 200	0.62	0.000	0.005
Apoptosis	157 / 161	0.53	0.000	0.005
E2F targets	181 / 200	0.66	0.002	0.006
Inflammatory response	194 / 200	0.62	0.004	0.006
Allograft rejection	192 / 200	0.74	0.002	0.006
PI3K Akt mTOR signaling	102 / 105	0.51	0.000	0.007
IL2 STAT5 signaling	189 / 199	0.54	0.004	0.008
G2m checkpoint	180 / 200	0.61	0.006	0.008
Myc targets v1	182 / 200	0.61	0.002	0.011
Epithelial mesenchymal transition	195 / 200	0.66	0.006	0.012
Hypoxia	187 / 200	0.47	0.000	0.012
Coagulation	135 / 128	0.50	0.006	0.013
KRAS signaling up	192 / 200	0.52	0.010	0.013
IL6 JAK STAT3 signaling	87 / 87	0.59	0.012	0.013
TNFA signaling via NFKB	193 / 200	0.55	0.021	0.015
Interferon alpha response	89 / 97	0.76	0.006	0.016
Unfolded protein response	106 / 113	0.47	0.002	0.018
MTORC1 signaling	189 / 200	0.52	0.012	0.020
Mitotic spindle	190 / 199	0.46	0.010	0.021
UV response up	150 / 158	0.40	0.000	0.027
Apical junction	192 / 200	0.44	0.004	0.029
Angiogenesis	36 / 36	0.57	0.020	0.037

[a] Hallmark gene sets; MSigDB, www.broadinstitute.org/gsea/msigdb

Supplementary Table 5. Top differentially expressed genes between 35P Q1 and Q4 in luminal A tumors (METABRIC Discovery cohort, n=208; * FDR < 0.001; ** FDR < 0.01; * FDR < 0.05)**

Gene	Ratio (Basal-like vs Luminal-like)	FDR[a]
MMP9	3.09	***
LOC652493	2.60	**
LOC647450	2.57	**
LOC652694	2.53	**
CXCL9	2.45	***
LOC649923	2.43	**
CXCL10	2.42	***
LYZ	2.30	***
LOC642113	2.30	*
RARRES1	2.10	***
S100A9	2.10	*
LOC651751	2.08	**
PITX1	2.02	*
CBX2	2.02	***
TUBB3	2.00	***
IGLL1	1.97	*
HS.534427	1.94	***
S100A8	1.93	*
CTHRC1	1.93	***
CD2	1.91	***
CD3D	1.89	***
COL10A1	1.89	**
EPSTI1	1.89	***
CXCR4	1.89	***
MMP11	1.88	**
CD52	1.86	**
CCL5	1.86	***
GZMK	1.84	**
SPOCK2	1.84	***
LOC649143	1.83	*
GBP1	1.83	***
POSTN	1.82	**
LOC652102	1.82	**
HLA-DQA1	1.82	***
MMP7	1.82	**
ISG15	1.81	**
HS.554324	1.80	***
IFI27	1.80	**
KRT80	1.79	***
FKSG30	1.78	***
CCL19	1.77	*
FABP5	1.76	***
HS.546375	1.75	***
MT2A	1.72	***
RAB11FIP1	1.72	***

IL7R	1.72	***
ISG20	1.72	***
FNDC1	1.72	**
BRF2	1.72	***
SULF1	1.71	***
CD79A	1.71	**
UBE2C	1.71	***
PLAU	1.71	***
KRT86	1.71	***
FPR3	1.70	***
GBP4	1.69	***
CD8A	1.69	***
LOC399942	1.69	***
ADAMDEC1	1.68	***
HLA-DRB4	1.67	**
APOC1	1.67	***
HCG4	1.67	***
C1QB	1.67	***
TNFSF13B	1.66	***
MGC29506	1.65	**
CD247	1.65	***
SFRP4	1.65	*
MX1	1.65	*
CD163	1.64	***
PTGDS	1.63	**
AGL	1.63	***
VAMP5	1.62	***
LGMN	1.62	***
SFRP2	1.62	**
CD48	1.62	***
PLA2G7	1.62	***
HLA-F	1.61	***
NNMT	1.61	***
HOPX	1.61	**
LTB	1.61	**
ITM2C	1.61	***
SQLE	1.61	***
CTSK	1.61	**
GJB2	1.61	*
SRGN	1.60	**
IGLL3	1.60	**
NKG7	1.60	***
CBLN2	1.59	*
GBP5	1.59	***
FAP	1.59	***
MT1E	1.59	*
CCL8	1.59	**
EYA2	1.59	***
RSAD2	1.58	***
UHRF1	1.58	***

KCNK1	1.58	*
LSM1	1.58	***
CTSC	1.58	***
RARRES2	1.58	**
HLA-DRB3	1.58	**
LOC402221	1.57	***
CDH11	1.57	***
SLAMF6	1.57	***
CTSL1	1.57	***
HLA-H	1.56	***
VCAN	1.56	**
LAPTM4B	1.56	***
ALG1L	1.56	**
HLA-B	1.56	***
GPNMB	1.56	**
DDHD2	1.56	***
MT1A	1.56	***
SLC7A5	1.55	**
C1ORF106	1.55	**
MFAP5	1.55	**
GGH	1.55	**
PVRIG	1.54	***
LOC647349	1.54	***
CDCA5	1.54	***
MS4A6A	1.54	**
FAM113B	1.54	***
C6ORF129	1.54	***
CXCL13	1.53	*
C1QC	1.53	**
LOC390354	1.53	***
LUM	1.53	*
C1S	1.53	**
PLTP	1.53	***
ADAM19	1.53	***
CCL4L2	1.53	**
IFI30	1.53	***
HERC6	1.53	**
NFS1	1.53	***
HLA-DRB6	1.53	**
TGM2	1.53	***
SERPINH1	1.52	***
GZMA	1.52	**
PRKCB1	1.52	***
ASH2L	1.52	***
COL5A1	1.52	*
ID3	1.52	***
TOP2A	1.52	*
PLAUR	1.51	***
PSMB9	1.51	***
STAT1	1.51	***

LAMP3	1.51	***
YBX1	1.51	***
LIME1	1.50	***
HCST	1.50	**
MYC	1.50	*
ALDH6A1	0.67	***
SEMA6A	0.67	*
FLJ41603	0.66	***
SPR	0.66	***
HSPA2	0.66	*
ARNT2	0.66	*
PPP4R4	0.66	**
HS.389988	0.66	*
HS.25318	0.66	**
MLPH	0.66	***
C1ORF21	0.66	***
TC2N	0.66	***
ACOT4	0.66	***
C4ORF32	0.66	***
ABCC8	0.66	*
GPD1L	0.65	***
PCM1	0.65	***
NEDD4L	0.65	***
SREBF1	0.65	**
OSBPL1A	0.65	***
CASC1	0.65	***
HIGD1A	0.65	***
C7ORF63	0.65	***
GSTO2	0.65	***
GPC4	0.65	**
CA12	0.65	**
AR	0.65	***
GLA	0.65	*
EVL	0.65	***
CHAD	0.64	*
TPSG1	0.64	*
ERP27	0.64	*
STEAP2	0.64	**
TMEM101	0.63	***
BNIPL	0.63	*
HS.570988	0.63	***
SYT9	0.63	***
C17ORF97	0.63	***
DYNLRB2	0.63	***
CTDSPL	0.63	***
SERPINA11	0.63	*
CGNL1	0.63	**
CYB5D2	0.63	***
IL17RB	0.62	**
KIAA1324	0.62	***

LOC145837	0.62	**
PDZK1IP1	0.62	***
SHRM	0.62	***
RASEF	0.62	**
ENTPD5	0.62	***
CYB5D1	0.62	***
C1ORF24	0.62	**
TMEM84	0.62	***
SERPINA3	0.61	*
TMEM26	0.61	**
LRRC48	0.61	***
SETBP1	0.61	***
NFIX	0.61	**
AGR3	0.61	*
DEGS2	0.61	***
GAD1	0.61	*
C6ORF97	0.61	***
GLRB	0.61	**
C14ORF45	0.61	***
ATP1B1	0.61	***
HS.125056	0.60	**
C6ORF126	0.60	**
INADL	0.60	***
ABCC11	0.60	**
CABYR	0.60	***
ENPP5	0.59	**
FAM129A	0.59	**
TMEM30B	0.59	***
RABEP1	0.59	***
LRIG1	0.59	***
PTPRT	0.59	**
SPAG6	0.59	**
SERHL	0.58	*
ELP2	0.58	***
EPHX2	0.58	***
PSD3	0.58	*
HS.561669	0.58	**
MUC1	0.58	***
VIPR1	0.58	***
ZNF533	0.58	*
IGSF21	0.57	**
LOC124220	0.57	***
GSTM3	0.57	***
GPR81	0.57	***
C1ORF218	0.56	**
NTN4	0.56	***
KIF13B	0.56	***
STC1	0.56	*
PPP1R3C	0.56	**
LYPD6	0.55	***

RTN1	0.55	*
SLC39A6	0.54	**
HS.134650	0.53	***
REEP6	0.53	***
STK32B	0.53	***
LOC389033	0.53	**
NKX3-1	0.53	**
LRP2	0.52	**
REEP1	0.52	***
SEC14L2	0.52	***
TGFBR3	0.52	***
LIMCH1	0.52	***
HS.159264	0.51	***
DIO1	0.51	**
TPRG1	0.50	***
LOC644844	0.50	**
KIF5C	0.50	***
ESR1	0.50	***
CYBRD1	0.50	***
SORBS2	0.49	***
NOSTRIN	0.49	***
HS.144479	0.47	**
GP2	0.46	**
FCGBP	0.46	**
TAT	0.45	*
PKIB	0.45	***
CFB	0.44	***
GRIA2	0.44	*
FMO5	0.43	***
LOC388743	0.43	***
STC2	0.42	**
IRX2	0.42	***
SCUBE2	0.42	***
NAT1	0.42	***
HMGCS2	0.41	*
CYP4Z2P	0.41	**
CYP4X1	0.40	**
CYP4Z1	0.40	*
LTF	0.37	**
LOC644151	0.32	***
AZGP1	0.31	***
PIP	0.04	***

[a] FDR = False discovery rate; Benjamini-Hochberg method

Supplementary Table 6. mRNA-protein correlation of 35P in the OSLO2 cohort[1] (Johansson et al. 2019)

35P	Correlation	p-value
TP53I3	0.688	< 0.001
LEPREL2	0.652	< 0.001
LSM1	0.633	< 0.001
NNMT	0.633	< 0.001
CBR1	0.630	< 0.001
PIP	0.603	< 0.001
PXDN	0.599	< 0.001
IDH2	0.598	< 0.001
WIPF1	0.551	< 0.001
CTSZ	0.540	< 0.001
MMP2	0.528	< 0.001
MYL9	0.504	< 0.001
ATP13A1	0.485	0.001
SEC24A	0.471	0.001
TOP1	0.395	0.008
TMSB4X	0.390	0.008
PSMC4	0.351	0.018
EFEMP2	0.308	0.040
VAT1	0.273	0.070
CDV3	0.261	0.083
AGRN	0.199	0.190
CMA1	0.192	0.205
EIF3F	0.130	0.395
PTRF	0.116	0.447
VWA1	0.109	0.473
ITIH1	0.064	0.676
LSM7	0.038	0.803
NID1	-0.228	0.133
FBF1	ND	ND
HLA-H	ND	ND
IGHG4	ND	ND
IGKV3D-15	ND	ND
IGKV3D-20	ND	ND
TMEM258	ND	ND
TPSAB1	ND	ND

ND: Not detected in OSLO2 mRNA dataset

Supplementary Table 7. Multivariate survival analysis of breast cancer specific deaths (proportional hazards regression model) stratified by molecular subtype (METABRIC Discovery cohort)

Variable	n	Univariate analysis		Multivariate analysis	
		HR (95 % CI)	p-value	HR (95 % CI)	p-value
**Luminal A subtype (n=466)**					
Tumor size					
< 20 mm	164	1.00	<0.001	1.00	**0.001**
≥ 20 mm	302	2.22 (1.44-3.42)		2.01 (1.36-3.25)	
Histologic grade					
1-2	333	1.00	0.010	1.00	0.090
3	133	1.63 (1.12-2.37)		1.39 (0.95-2.03)	
Lymph node status					
Negative	273	1.00	0.014	1.00	0.054
Positive	193	1.58 (1.10-2.27)		1.44 (0.95-2.08)	
35P Stromal signature					
Q1-Q3	426	1.00	0.002	1.00	**0.002**
Q4	40	2.25 (1.36-3.72)		2.27 (1.36-3.77)	
**Luminal B subtype (n=268)**					
Tumor size					
< 20 mm	64	1.00	0.005	1.00	0.083
≥ 20 mm	204	1.99 (1.24-3.20)		1.57 (0.94-2.60)	
Histologic grade					
1-2	102	1.00	0.099	1.00	0.540
3	166	1.38 (0.94-2.03)		1.13 (0.76-1.69)	
Lymph node status					
Negative	127	1.00	<0.001	1.00	**<0.001**
Positive	141	2.39 (1.63-3.51)		2.10 (1.41-3.14)	
35P Stromal signature					
Q1-Q3	199	1.00	0.464	1.00	0.384
Q4	69	1.17 (0.77-1.76)		1.21 (0.79-1.85)	
**Basal-like subtype (n=118)**					
Tumor size					
< 20 mm	35	1.00	0.753	1.00	0.293
≥ 20 mm	83	0.91 (0.49-1.68)		0.71 (0.38-1.34)	
Histologic grade					
1-2	8	1.00	0.324	1.00	0.519
3	110	2.04 (0.50-8.42)		1.60 (0.38-6.71)	
Lymph node status					
Negative	53	1.00	0.006	1.00	**0.005**
Positive	65	2.39 (1.28-4.48)		2.62 (1.35-5.11)	
35P Stromal signature					
Q1-Q3	14	1.00	0.992	1.00	0.373
Q4	104	0.96 (0.41-2.26)		0.67 (0.27-1.63)	

CI: Confidence interval. HR: hazard ratio. n: number of patients. NS: not significant.

Supplementary Table 8. Multivariate survival analysis of IntClust and 35P with tumor size, histologic grade and lymph node status as confounding factors.

Variable	N	HR vs reference (95 % CI)	p-value
**Tumor size**			
< 20 mm vs ≥ 20 mm	263/ 589	1.67 (1.24-2.24)	< 0.001
**Histologic grade**			
1-2 vs 3	443 / 409	1.40 (1.07-1.84)	0.015
**Lymph node status**			
Negative vs Positive	453 / 399	1.69 (1.32-2.17)	< 0.001
**35P**			
Q1-Q3 vs Q4	639 / 219	1.58 (1.15-2.16)	0.004
**IntClust**			
Global[a]	852	NA	< 0.001

[a] IntClust (clusters 1–10) was included as a factor in the multivariable Cox model. The p-value shown is from a global Wald test for the variable overall; hazard ratios for individual clusters versus the reference are not shown.

Supplementary Table 9. **Multivariate analysis of 35P score and distant recurrence free interval (DRFI) in patients from the STO trials**

	35P score Tertile cutoff[fa]	Patients No.	DR [c] 20yr No.	aHR[b] (95% CI) DRFI[c]	35P score Quartile cutoff[a]	Patients No.	DR[c] 20yr No.	aHR[b] (95% CI) DRFI[c]
ER+ HER2- patients	T12	662	207	1.00 (ref.)	Q123	743	244	1.00 (ref.)
	T3	332	120	1.21 (0.95–1.55)	Q4	251	83	1.12 (0.85–1.46)
Control	T12	285	106	1.00 (ref.)	Q123	326	128	1.00 (ref.)
	T3	143	63	1.24 (0.88–1.74)	Q4	102	41	1.23 (0.84–1.80)
Tamoxifen	T12	377	101	1.00 (ref.)	Q123	417	116	1.00 (ref.)
	T3	189	57	1.38 (0.96–1.99)	Q4	149	42	1.17 (0.78–1.75)
Luminal A patients	T12	437	117	1.00 (ref.)	Q123	490	134	1.00 (ref.)
	T3	214	56	1.21 (0.86–1.71)	Q4	161	39	1.14 (0.78–1.67)
Control	T12	194	63	1.00 (ref.)	Q123	218	73	1.00 (ref.)
	T3	91	30	1.47 (0.89–2.41)	Q4	67	20	1.35 (0.78–2.34)
Tamoxifen	T12	243	54	1.00 (ref.)	Q123	272	61	1.00 (ref.)
	T3	123	26	1.04 (0.61–1.76)	Q4	94	19	1.03 (0.58–1.82)
Luminal B Patients	T12	157	72	1.00 (ref.)	Q123	179	90	1.00 (ref.)
	T3	97	55	1.21 (0.80–1.81)	Q4	75	37	1.02 (0.67–1.57)
Control	T12	63	34	1.00 (ref.)	Q123	77	45	1.00 (ref.)
	T3	44	30	0.84 (0.45–1.58)	Q4	30	19	1.14 (0.61–2.11)
Tamoxifen	T12	94	38	1.00 (ref.)	Q123	102	45	1.00 (ref.)
	T3	53	25	**2.08 (1.07–4.02)**	Q4	45	18	1.27 (0.61–2.62)

[a] Two cutoffs are presented. Tertile cutoff at the upper tertile (T3) and quartile cutoff at the upper quartile (Q4) versus the rest.

[b] aHR: adjusted Hazard Ratio. Multivariable Cox proportional hazard regression adjusted for age, menopausal status, random assignment period, tumor size, tumor grade, lymph node status, PR status, Ki67-status, type of surgery, chemotherapy and radiotherapy status.

In addition: models in the overall ER+/HER2− cohort included tamoxifen therapy and PAM50 subtype; ER+/HER2− models stratified by treatment (control vs tamoxifen) included PAM50 subtype but not tamoxifen therapy; models in the overall Luminal A / Luminal B cohorts included tamoxifen therapy but not PAM50 subtype; models restricted to tamoxifen-treated Luminal A / Luminal B patients did not additionally adjust for tamoxifen or PAM50 subtype.

[C] DR: Distant recurrence; DRFI: Distant recurrence-free interval

Supplementary Table 10. IMC antibody panel

Metal	Target	Clone	Cat no	Supplier
141Pr	aSMA	1A4	3141017D-DVS	Fluidigm
142Nd	Cytokeratin 5/6	E2T4B	81817	CST
143Nd	Vimentin	D21H3	3143027D	Fluidigm
144Nd	p53	DO-7	48818BF	CST
145Nd	VAChT	EPR24248-167	ab279710	Abcam
146Nd	MAP2	SP88	ab240935	CST
147Sm	CD163	EDHu-1	3147021D-DVS	Fluidigm
148Nd	Pan-Cytokeratin	C11/AE1/AE3	3148022D	Fluidigm
149Sm	Stathmin	D1Y5A	13655BF	CST
150Nd	Sox2	EPR3131	ab215970	Abcam
151Eu*	CD31	ICO115	3569BF	CST
151Eu*	CD34	EPR3094	3151025D	Fluidigm
152Sm	CD45	D9M8I	3152018D	Fluidigm
153Eu	CD44	IM7	3153029D-DVS	Fluidigm
154Sm	TH	E2L6M	58844BF	CST
155Gd	FOXP3	D6O8R	12653BF	CST
156Gd	CD4	EPR6855	3156033D-DVS	Fluidigm
158Gd	NCAM	E7X9M	99746BF	CST
159Tb	CD68	KP1	3159035D-DVS	Fluidigm
160Gd	Nestin	10C2	656802	Biolegend
161Dy	CD20	H1	3161029D-DVS	Fluidigm
162Dy	CD8	C8/144B	3162034D-DVS	Fluidigm
163Dy	ER	SP1	ab187260	Abcam
164Dy	Cytokeratin 14	SP53	ab236439	Abcam
165Ho	PD1	EPR4877(2)	3165039D-DVS	Fluidigm
166Er	HER2	D8F12	4290BF	CST
167Er	GATA3	D13C9	5852BF	CST
168Er	Ki67	B56	3168022D-DVS	Fluidigm
169Tm	PDPN	LpMab-12	26981BF	CST
170Er	CD3	Polyclonal	3170019D-DVS	Fluidigm
171Yb	PDGFRb	28E1	3169BF	CST
172Yb	Peripherin	Polyclonal	NBP2-38444	Biotechne
173Yb	DCX	EPR19997	ab222921	Abcam
174Yb	Cytokeratin 8/18	C51	3174022D	Fluidigm
175Lu	Neurofilament	C28E10	2837BF	Abcam
176Yb	H3	D1H2	3176023D-DVS	Fluidigm

*CD31 and CD34 share the same metal (151Eu)

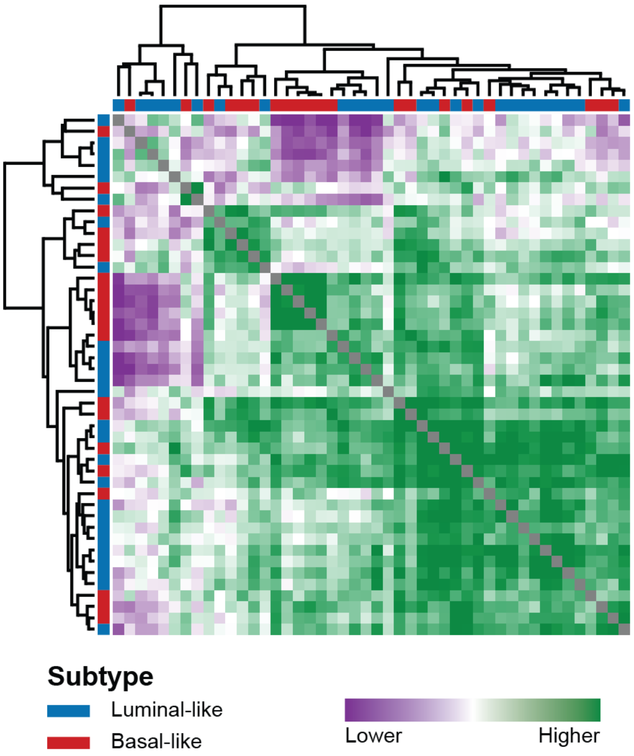

Supplementary Figure 1. Correlation plot of breast cancer whole tissue samples.

Unsupervised hierarchical clustering of global proteome correlation data between whole tissue breast cancer samples (basal-like, n= 20; luminal-like, n=26).

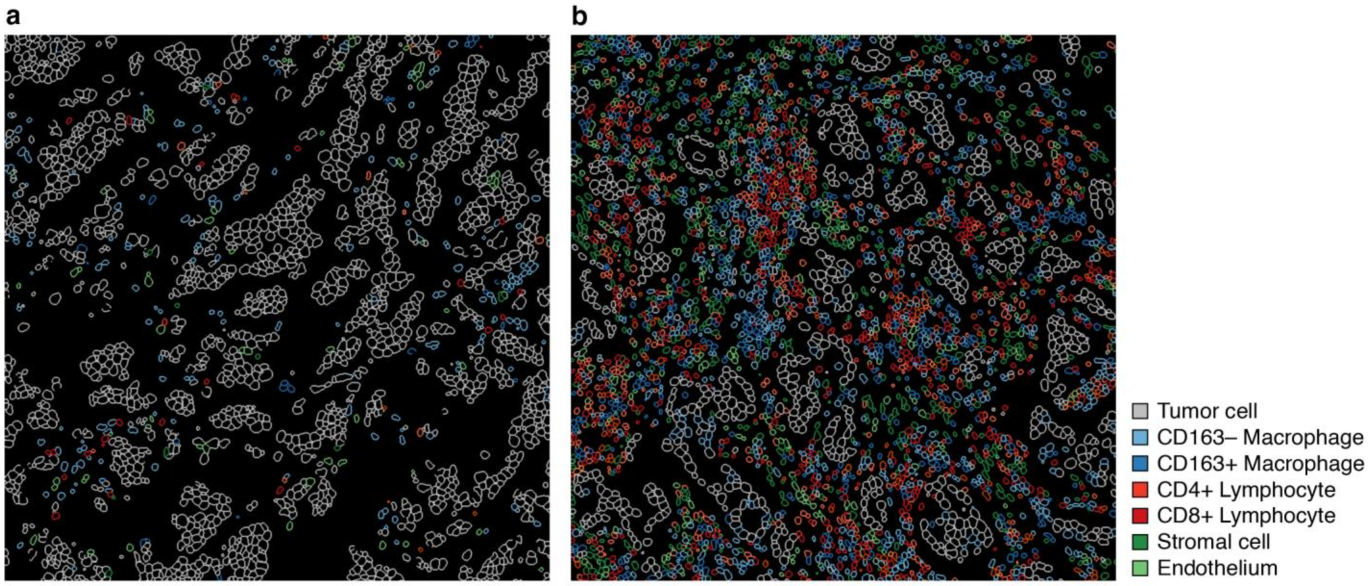

Supplementary Figure 2. Masks from Imaging Mass Cytometry (IMC) data.

Representative areas from low (**a**) and high (**b**) tumor-stromal cellularity. Both images are 850x850 pixels.

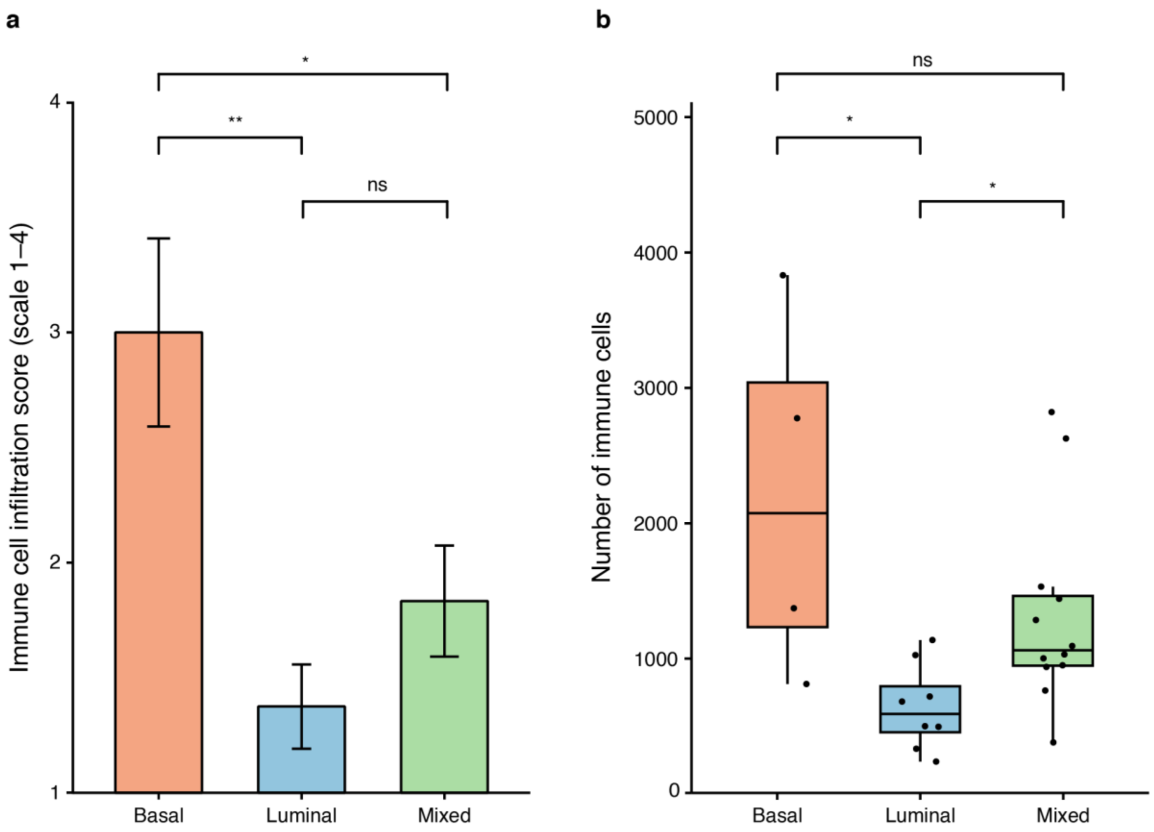

Supplementary Figure 3. Immune cell infiltration score in the three stromal subgroups (after un-supervised clustering).

The tumor stroma of each of the 24 laser microdissected cases were scored according to immune cell infiltration status: 1 = no infiltration; 2 = low infiltration; 3 = moderate infiltration; 4 = considerable infiltration (**a**); and using immune cell counts (T-cells, B-cells and macrophages) from in-house Imaging Mass Cytometry data (**b**). The difference between groups was tested using Mann-Whitney U-test; ** $p<0.01$; * $p<0.05$; ns= not significant.

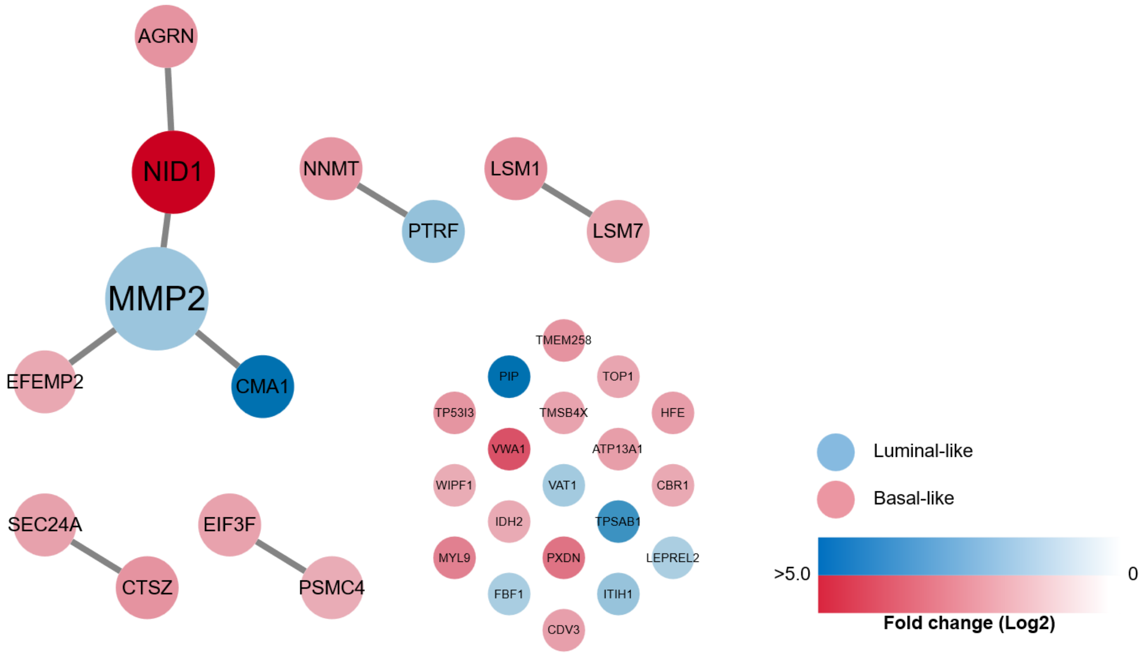

Supplementary Figure 4. STRING-generated network of the 35 proteins (35P) in the stromal proteome signature.

Protein-protein interaction network of the 35P stromal signature proteins. The color intensity of the nodes represents fold change (Log_2) between the luminal-like subtype (blue) and basal-like subtype (red).

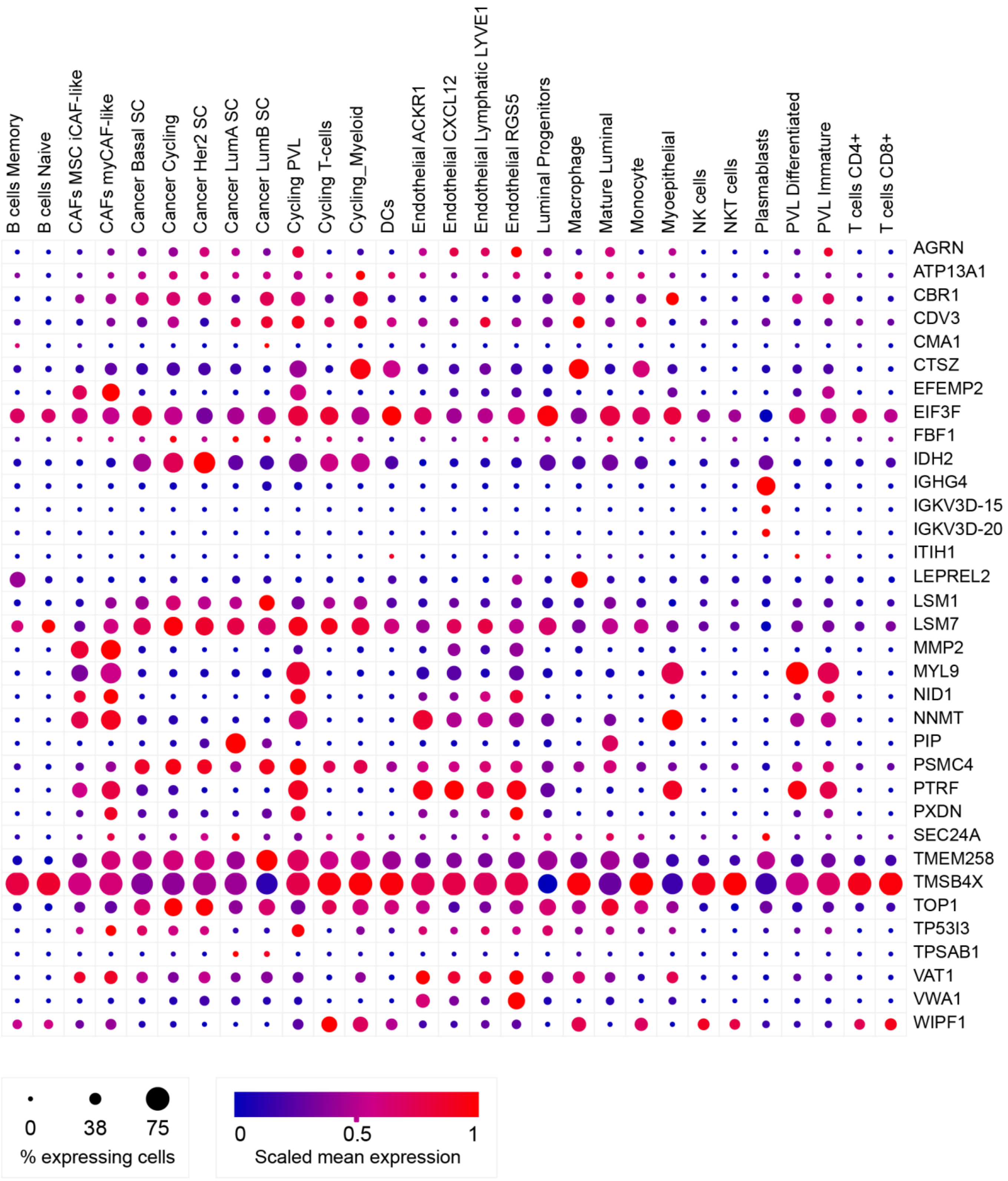

Supplementary Figure 5. Overview of cell types expressing each of the 35P proteins.

Single-cell data from Wu et al. (2021)[2], accessed via singlecell.broadinstitute.org. Each 35P protein was queried using the entire breast cancer atlas, with "celltype_minor"-annotations. Subsampling was set to 100,000. Dot size represents relative proportion of annotated cells with expression of marker. Red and blue colors show the scaled mean expression of the marker. CAF – cancer associated fibroblast; MSC – mesenchymal stem cell; SC – single-cell derived intrinsic subtype; PVL – perivascular-like; DC – dendritic cell; NK – natural killer.

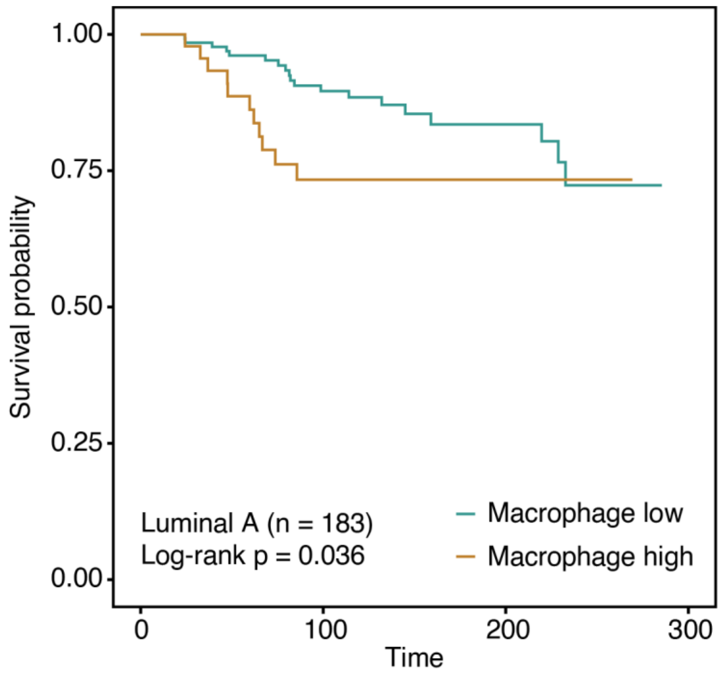

Supplementary Figure 6. Univariate survival analysis (Kaplan-Meier method; breast cancer specific deaths) of luminal A cases (n=183) with high (upper quartile) and low (the rest) macrophage count (METABRIC cohort).

The plot shows an association between high macrophage count and poor survival. Data is collected from Danenberg et al. single-cell study of cases from the METABRIC cohort[3].

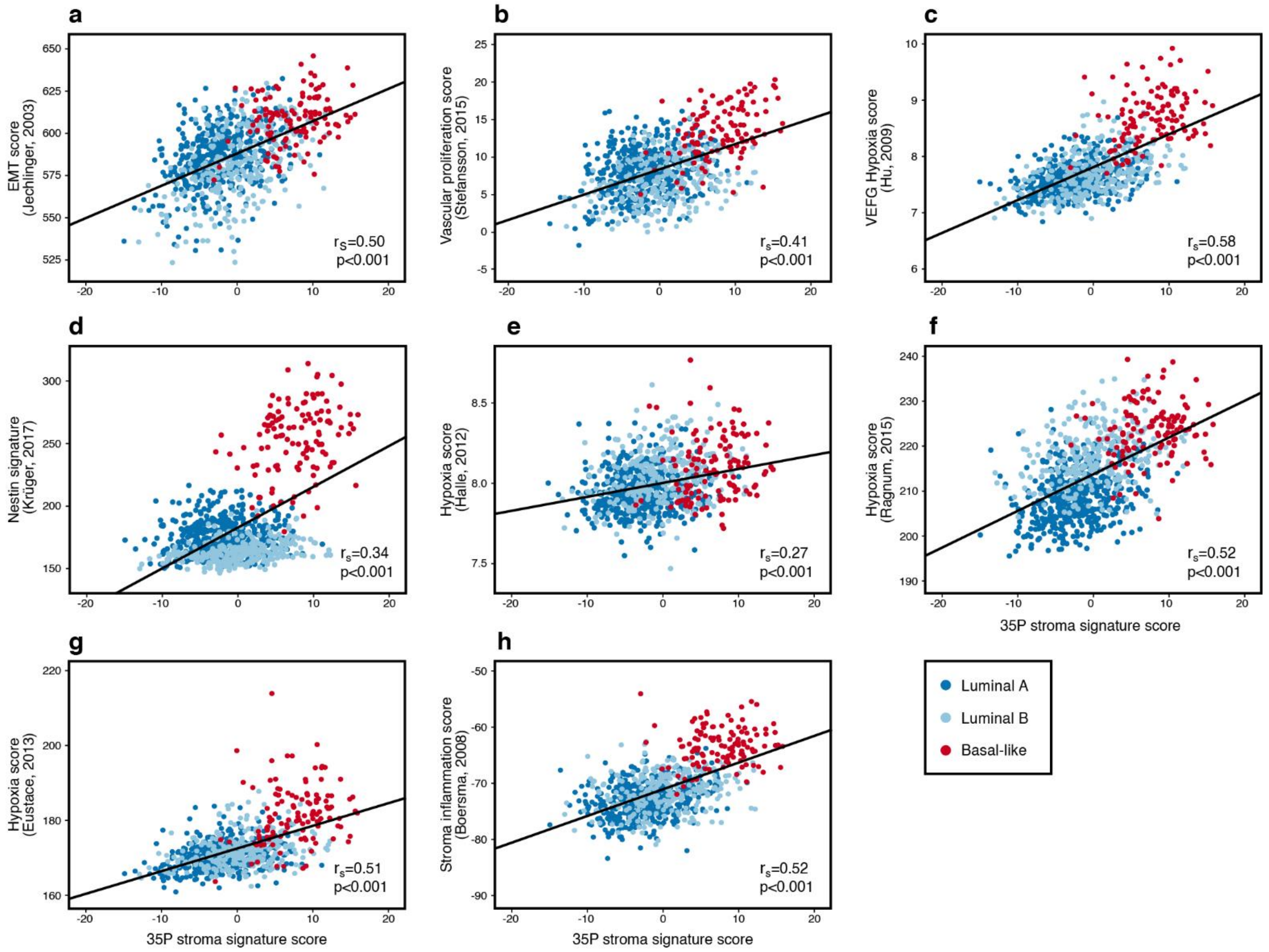

Supplementary Figure 7. The 35P signature correlates to features (by gene expression signatures) associated with high-grade tumors (METABRIC cohort).

The 35P stromal signature scores were plotted against scores from various gene expression signatures associated with high grade tumors. Significant positive correlations were seen for EMT[4] (**a**), angiogenesis[5,6] (**b-c**), stemness[7] (**d**), hypoxia[8-10] (**e-g**) and stromal inflammation[11] (**h**).

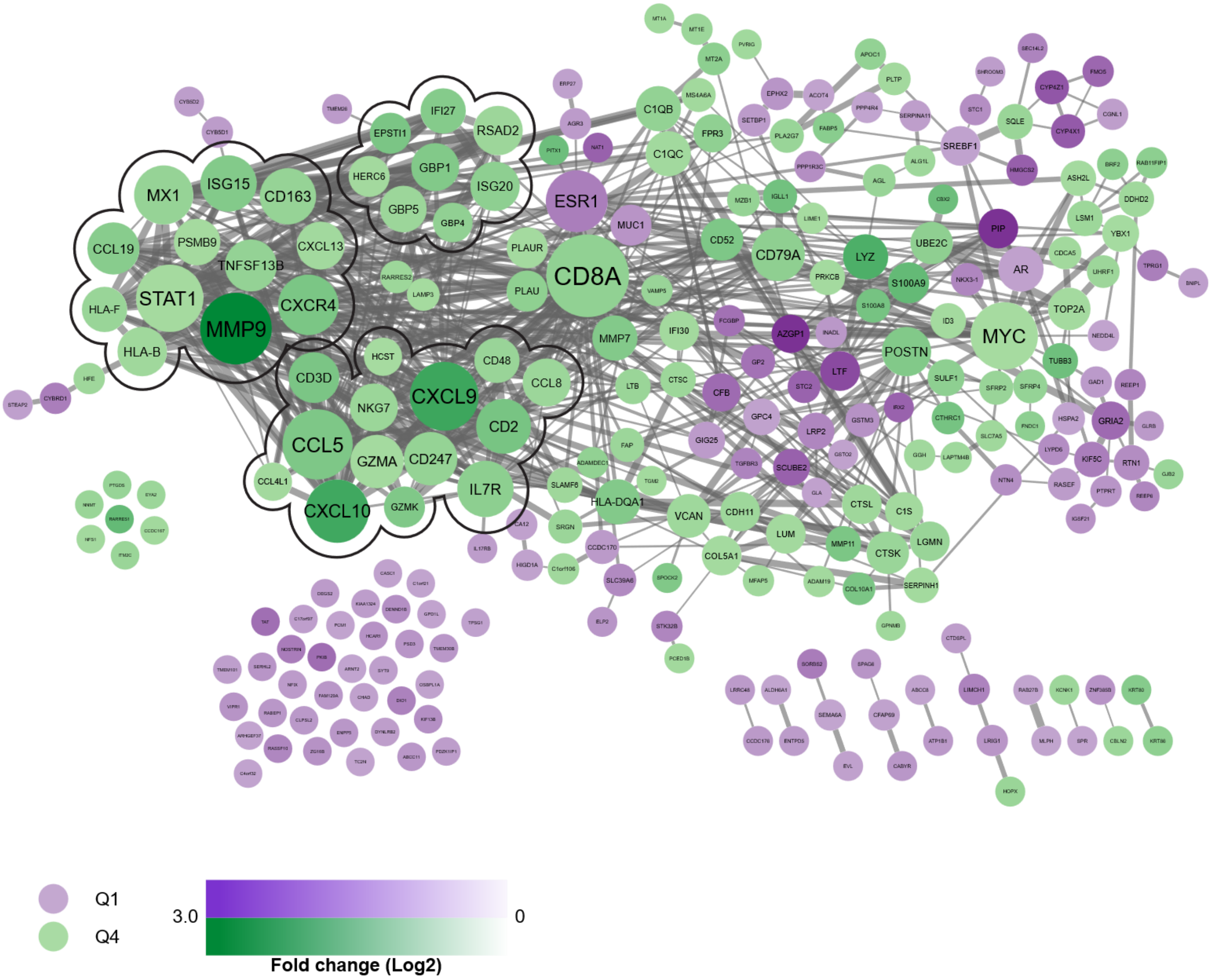

Supplementary Figure 8. Interaction network of genes differentially expressed between 35P-high (Q4) and 35P-low (Q1) luminal A tumors.

Protein-protein association network of 287 corresponding proteins (by their mRNA values) significantly differentially expressed (FDR<0.05, FC>1.5) between 35P-high (Q4) (green) and 35P-low (Q1) (purple) in patients with luminal A breast cancer (METABRIC Discovery cohort; n=466) (PPI enrichment p-value<1.0×10^{-16}). The color intensity of nodes represents fold change. Encircled protein clusters represent sub-networks identified by the MCODE Cytoscape addon.

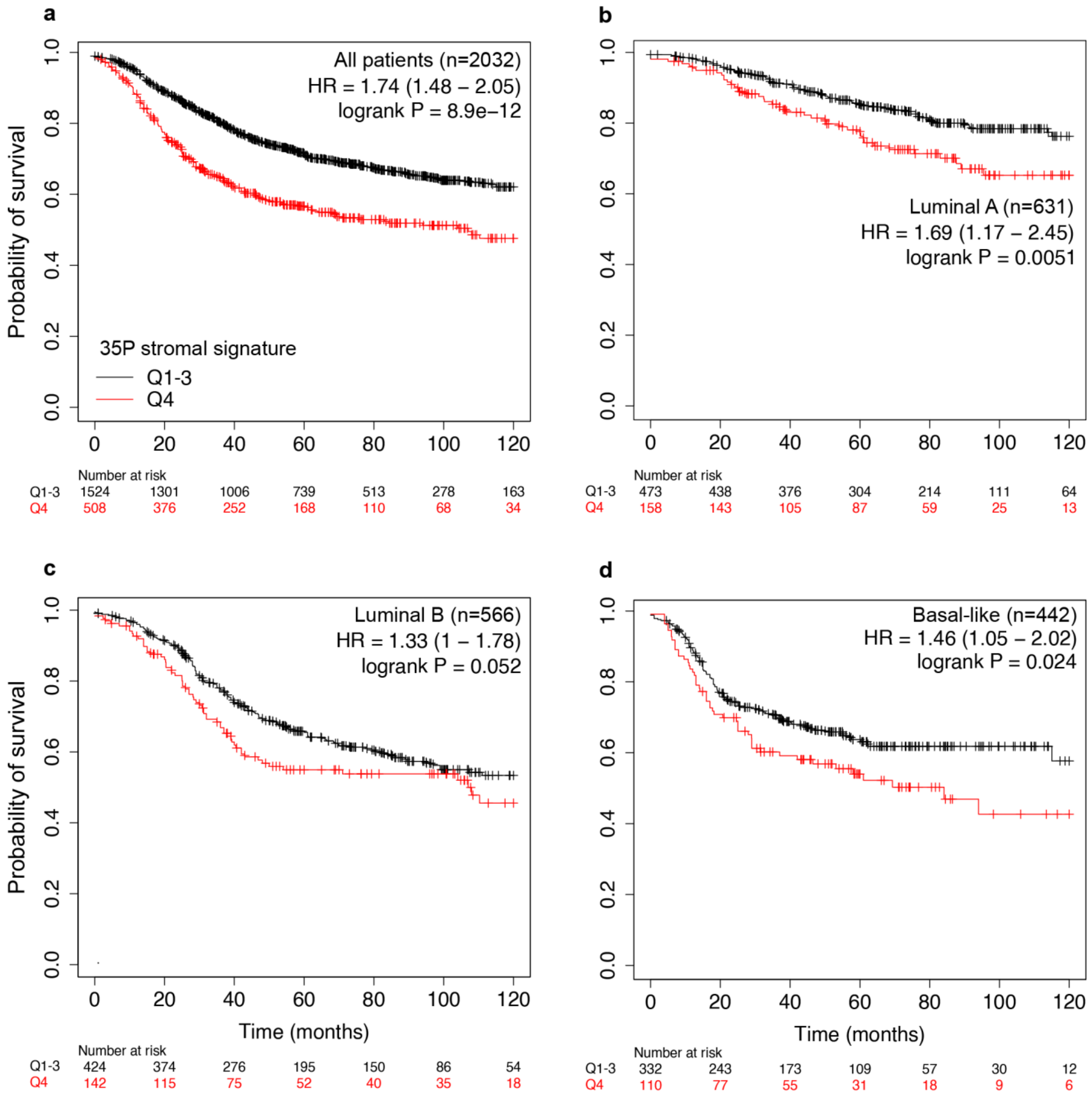

Supplementary Figure 9. Survival analyses of the 35P stromal signature (KMPlotter).

Kaplan-Meier plots were generated by KMPlotter[12] with 35P-stromal signature as input, and by separating patients by upper quartile. Recurrence-free survival in all patients (no restrictions) included 2032 patients (**a**). The patients were stratified into luminal A (**b**), luminal B (**c**), and basal-like (**d**) subtypes (by PAM50). The analyses were truncated at 10-years follow-up.

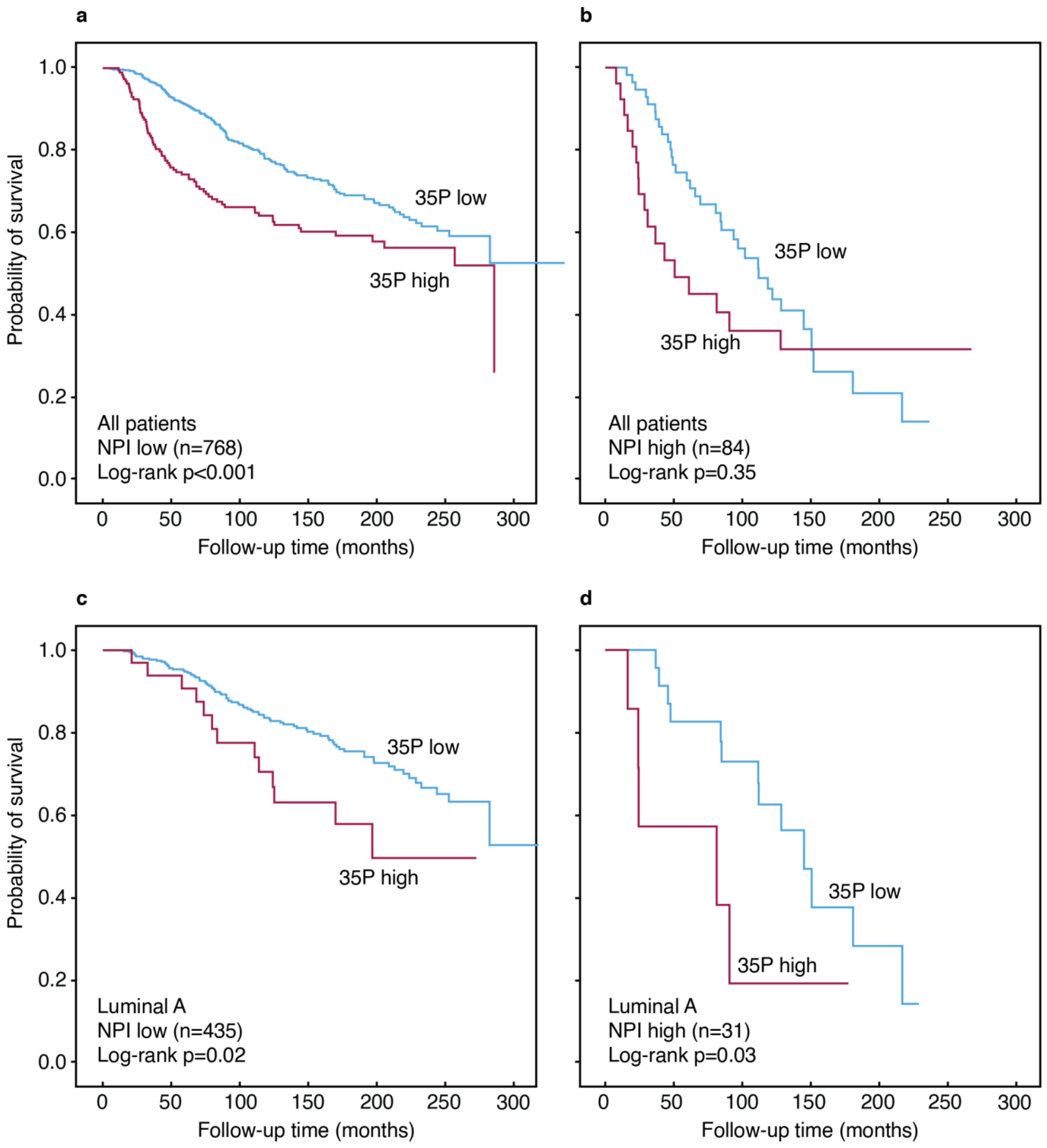

Supplementary Figure 10. Univariate survival analysis (Kaplan-Meier method; breast cancer specific deaths) of 35P (upper quartile vs the rest) in all patients (n = 852) and luminal A (n=466) stratified by NPI-low and NPI-high (METABRIC cohort).

Among all patients, high 35P is associated with poor survival in NPI-low (**a**), but not in NPI-high (**b**). In patients with luminal A breast cancer, 35P is associated with poor survival in both NPI-low (**d**) and NPI-high (**d**) groups.

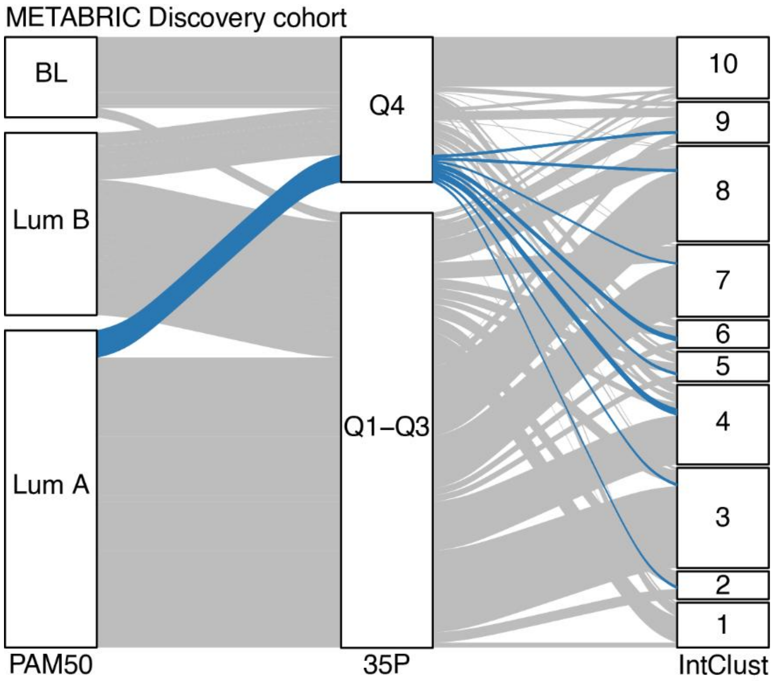

Supplementary Figure 11. Alluvial plot with PAM50, 35P and IntClust subgroups (METABRIC Discovery cohort).

The plot shows how the 35P-high (Q4) luminal A cases (shown in blue) are distributed across the IntClust subgroups.

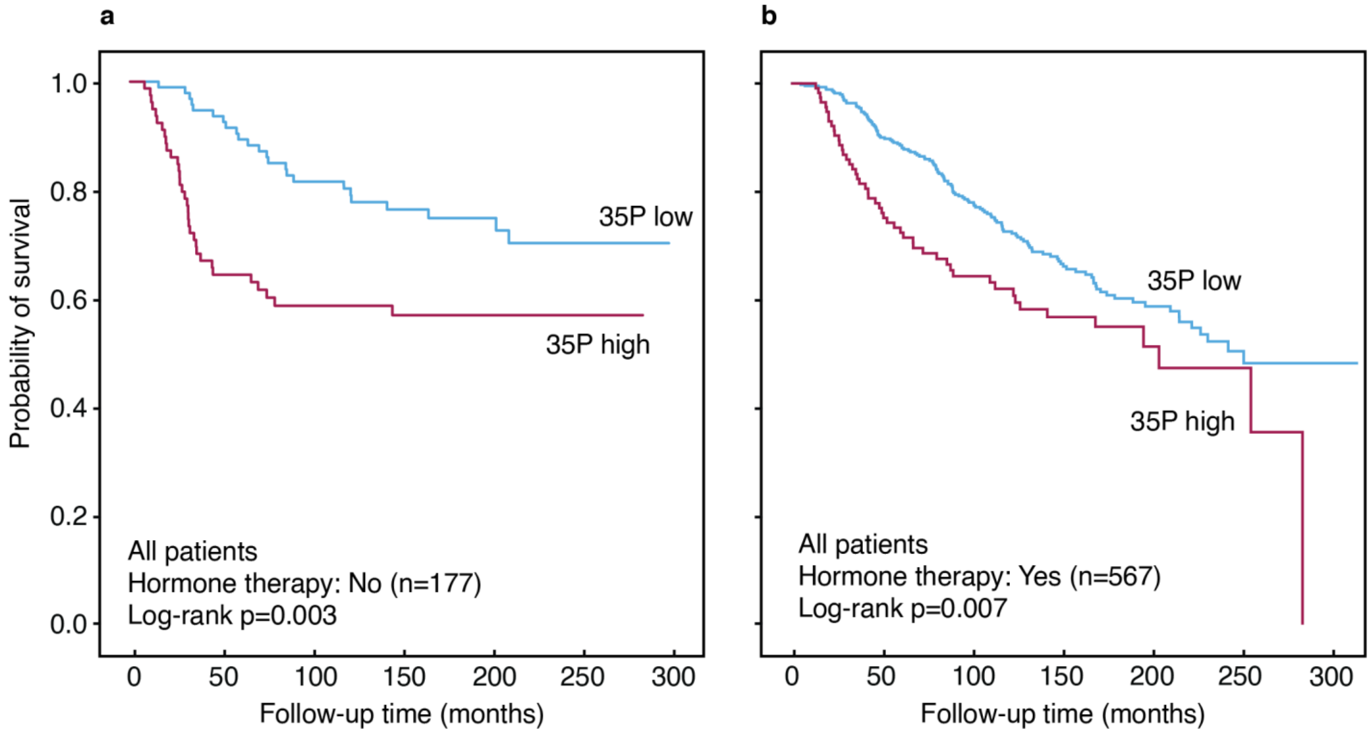

Supplementary Figure 12. Univariate survival analysis (Kaplan-Meier method; breast cancer specific deaths) of 35P high (upper quartile) versus 35P low (the rest), stratified by hormone therapy (METABRIC cohort).

All patients with information on hormone therapy were included (n = 744). Endpoint is breast cancer specific survival. The plots show a significant association between 35P-high and survival in patients without hormone therapy (a) and with hormone therapy (b).

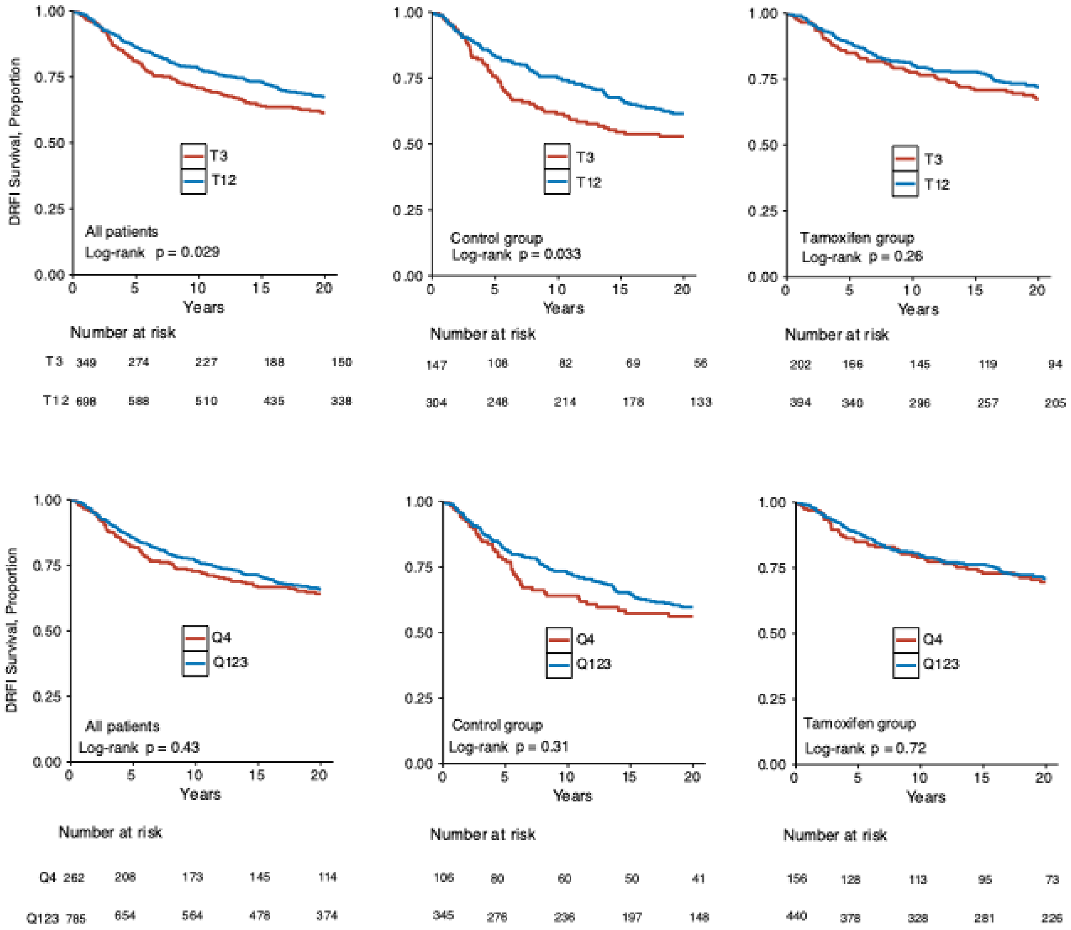

Supplementary Figure 13. Univariate long-term survival of ER+/HER2– breast cancer patients from the STO trials.

Two cutoffs are presented with **Distant Recurrence-Free interval (DRFI)** of all patients (n=1,047), control group (n=451) and tamoxifen group (n=596). Tertile cutoff at the upper tertile (T3) **(a-c)** and quartile cutoff at the upper quartile (Q4) **(d-f)** versus the rest.

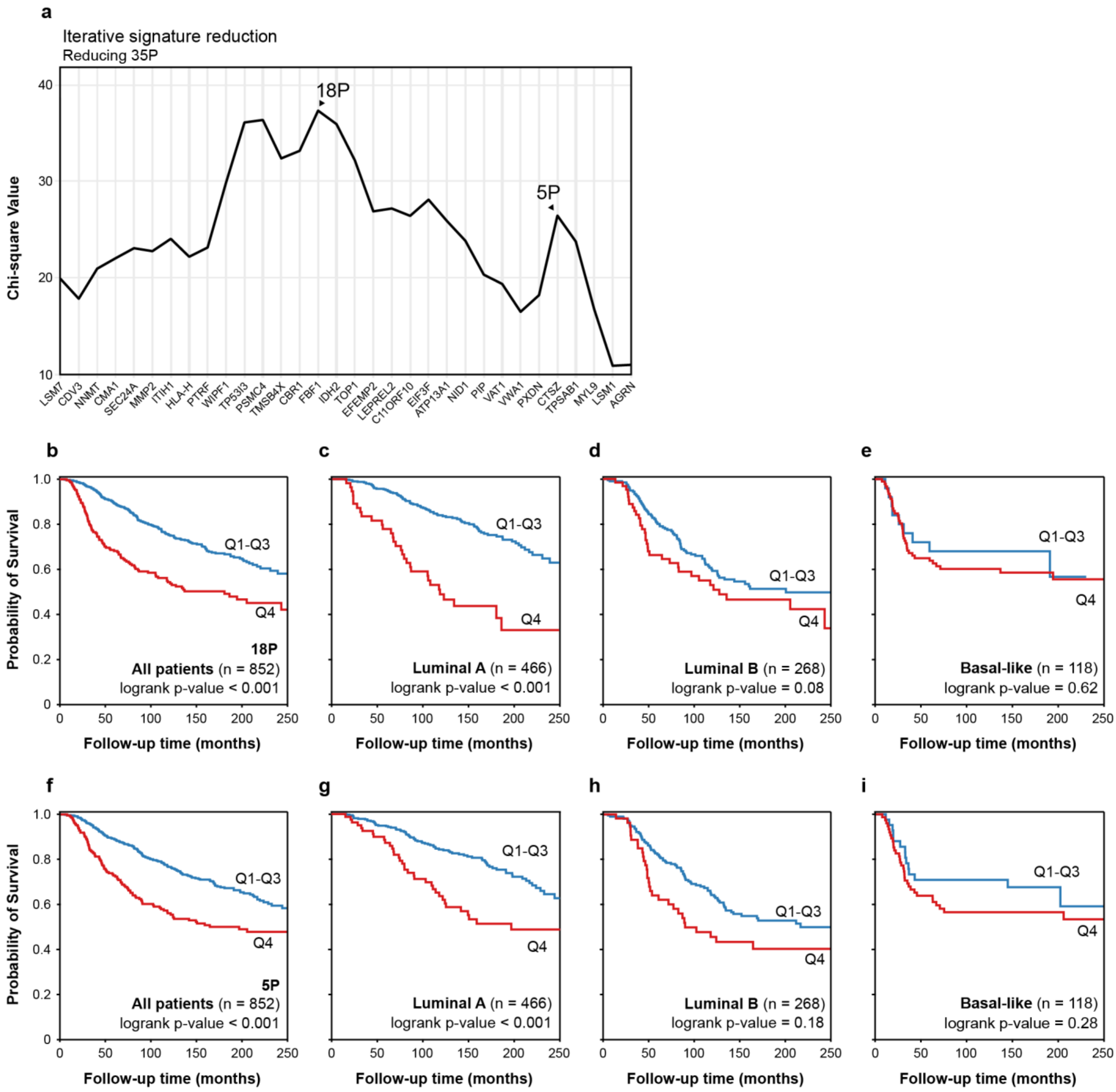

Supplementary Figure 14. Iterative signature reduction analysis.

One protein at a time was iteratively removed from 35P, and the resulting subset was tested by the log-rank test. The best performing protein subset was retained, and the process was repeated until only one protein remained. The ‘peak’ (i.e. highest chi-square value) was reached by a subset of 18-proteins (**a**). Another peak was reached by a subset of 5-proteins. Univariate survival analyses of 18P and 5P in all patients, luminal A, luminal B and basal-like subtypes in the METABRIC Discovery cohort (**b-i**).

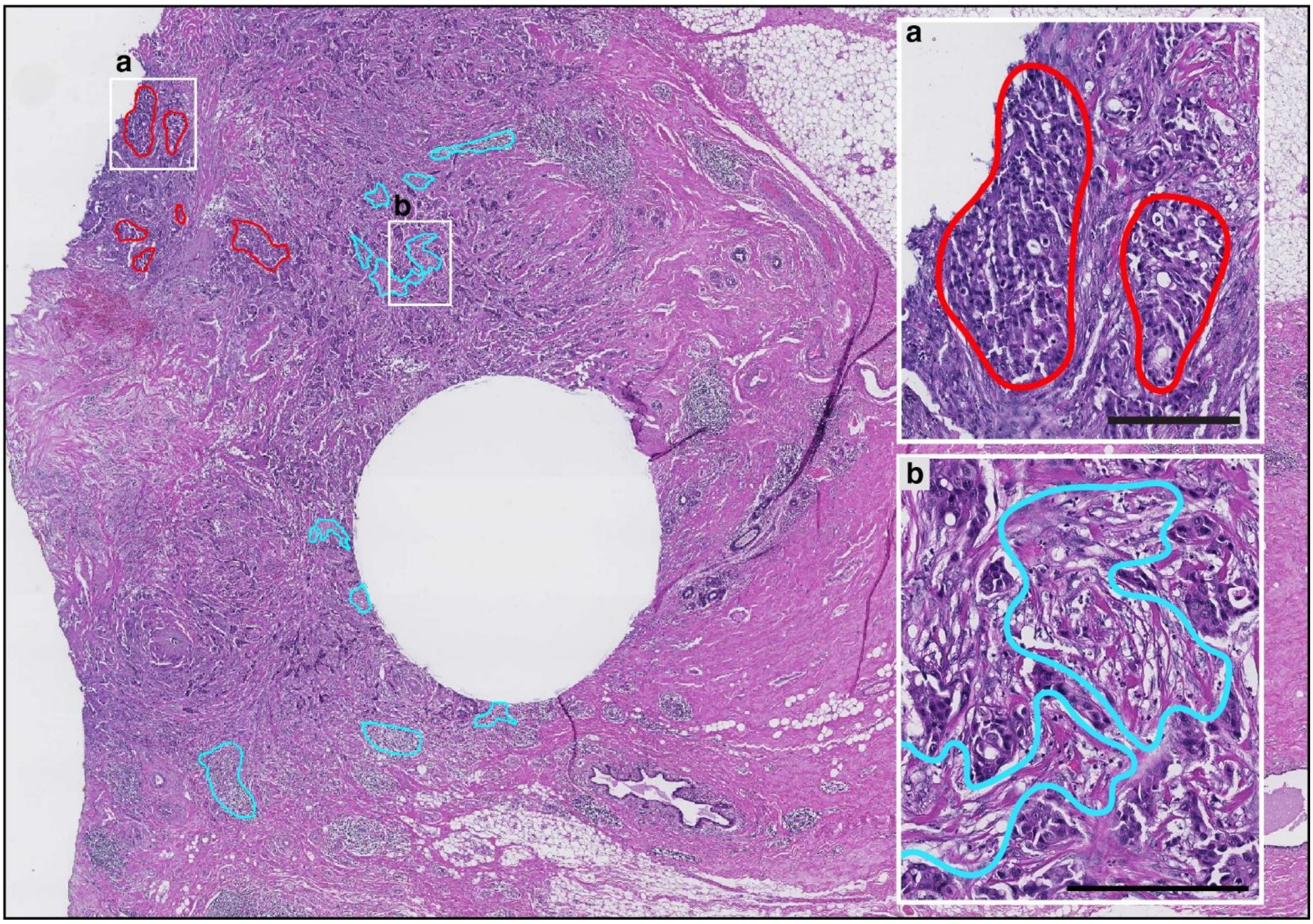

Supplementary Figure 15. A representative image of breast cancer tissue included for laser-capture microdissection (LCM).

Areas in red are tumor cells (a), and areas in light blue are stromal areas (b) selected for LCM. The large white circle in the center represents an area of tumor tissue removed to build a tumor microarray (TMA).

References

1 Johansson, H. J. *et al.* Breast cancer quantitative proteome and proteogenomic landscape. *Nat Commun* **10**, 1600 (2019). https://doi.org/10.1038/s41467-019-09018-y

2 Wu, S. Z. *et al.* A single-cell and spatially resolved atlas of human breast cancers. *Nat Genet* **53**, 1334-1347 (2021). https://doi.org/10.1038/s41588-021-00911-1

3 Danenberg, E. *et al.* Breast tumor microenvironment structures are associated with genomic features and clinical outcome. *Nat Genet* **54**, 660-669 (2022). https://doi.org/10.1038/s41588-022-01041-y

4 Jechlinger, M. *et al.* Expression profiling of epithelial plasticity in tumor progression. *Oncogene* **22**, 7155-7169 (2003). https://doi.org/10.1038/sj.onc.1206887

5 Stefansson, I. M. *et al.* Increased angiogenesis is associated with a 32-gene expression signature and 6p21 amplification in aggressive endometrial cancer. *Oncotarget* **6**, 10634-10645 (2015). https://doi.org/10.18632/oncotarget.3521

6 Hu, Z. *et al.* A compact VEGF signature associated with distant metastases and poor outcomes. *BMC Med* **7**, 9 (2009). https://doi.org/10.1186/1741-7015-7-9

7 Kruger, K. *et al.* Expression of Nestin associates with BRCA1 mutations, a basal-like phenotype and aggressive breast cancer. *Sci Rep* **7**, 1089 (2017). https://doi.org/10.1038/s41598-017-00862-w

8 Halle, C. *et al.* Hypoxia-induced gene expression in chemoradioresistant cervical cancer revealed by dynamic contrast-enhanced MRI. *Cancer research* **72**, 5285-5295 (2012). https://doi.org/10.1158/0008-5472.CAN-12-1085

9 Ragnum, H. B. *et al.* The tumour hypoxia marker pimonidazole reflects a transcriptional programme associated with aggressive prostate cancer. *Br J Cancer* **112**, 382-390 (2015). https://doi.org/10.1038/bjc.2014.604

10 Eustace, A. *et al.* A 26-gene hypoxia signature predicts benefit from hypoxia-modifying therapy in laryngeal cancer but not bladder cancer. *Clinical cancer research : an official journal of the American Association for Cancer Research* **19**, 4879-4888 (2013). https://doi.org/10.1158/1078-0432.CCR-13-0542

11 Boersma, B. J. *et al.* A stromal gene signature associated with inflammatory breast cancer. *International journal of cancer. Journal international du cancer* **122**, 1324-1332 (2008). https://doi.org/10.1002/ijc.23237

12 Gyorffy, B. *et al.* An online survival analysis tool to rapidly assess the effect of 22,277 genes on breast cancer prognosis using microarray data of 1,809 patients. *Breast Cancer Res Treat* **123**, 725-731 (2010). https://doi.org/10.1007/s10549-009-0674-9
